# Characterising the harmonic vocal repertoire of the Indian wolf (*Canis lupus pallipes*)

**DOI:** 10.1371/journal.pone.0216186

**Published:** 2019-10-31

**Authors:** Sougata Sadhukhan, Lauren Hennelly, Bilal Habib

**Affiliations:** 1 Department of Animal Ecology and Conservation Biology, Wildlife Institute of India, Dehradun, India;; 2 Mammalian Ecology and Conservation Unit, Veterinary Genetics Laboratory, University of California Davis, Davis, California, United States of America; Centre for Cellular and Molecular Biology, INDIA

## Abstract

Vocal communication in social animals plays a crucial role in mate choice, maintaining social structure, and foraging strategy. The Indian grey wolf, among the least studied subspecies, is a social carnivore that lives in groups called packs and has many types of vocal communication. In this study, we characterise harmonic vocalisation types of the Indian wolf using howl survey responses and opportunistic recordings from captive and nine packs (each pack contains 2–9 individuals) of free-ranging Indian wolves. Using principal component analysis, hierarchical clustering, and discriminant function analysis, we found four distinct vocalisations using 270 recorded vocalisations (Average Silhouette width Si = 0.598) which include howls and howl-barks (N = 238), whimper (N = 2), social squeak (N = 28), and whine (N = 2). Although having a smaller body size compared to other wolf subspecies, Indian wolf howls have an average mean fundamental frequency of 422 Hz (±126), which is similar to other wolf subspecies. The whimper showed the highest frequency modulation (37.296±4.601) and the highest mean fundamental frequency (1708±524 Hz) compared to other call types. Less information is available on the third vocalisation type, i.e. ‘Social squeak’ or ‘talking’ (Mean fundamental frequency = 461±83 Hz), which is highly variable (coefficient of frequency variation = 18.778±3.587). Lastly, we identified the whine, which had a mean fundamental frequency of 906Hz (±242) and is similar to the Italian wolf (979±109 Hz). Our study’s characterisation of the Indian wolf’s harmonic vocal repertoire provides a first step in understanding the function and contextual use of vocalisations in this social mammal.

## Introduction

Vocalisation plays a critical role in social animals for conveying information on foraging, reproductive, and social behaviours [1–7]. Characterising the vocal repertoire of a species provides a base for understanding the behavioural significance of different vocalisations and studying how vocal communication varies across populations, subspecies, and taxa [8–10].

The wolf (*Canis lupus*) is a social mammal and uses a variety of vocalisations for communication. Being present throughout Eurasia and North America, the wolf is one of the most widely distributed land mammals and occupies a wide range of different habitat types [11]. While there has been much research on wolves in North America and Europe, much less has been done on the wolves of Asia. For the grey wolf, most of the mitochondrial diversity is centred in southern and central Asia, where two independent and phylogenetically basal maternal lineages–the Tibetan and Indian wolf–are found [12–14]. The Tibetan and Indian wolf maternal lineages are estimated to have diverged around 700,000, and 300,000 years ago, respectively [12,15,16]. Despite its phylogenetic position as one of the oldest maternal lineages and among the smallest subspecies [12], relatively little is known about Indian wolf ecology and behaviour compared to other wolf subspecies. Studying the vocalisations of the Indian wolf can offer a greater understanding of the behavioural function of different vocal signals in Indian wolves and, more broadly, the variation in vocalisation and associated behaviour across subspecies and taxa within the *Canis* clade.

The best-known wolf vocalisation–the howl–is a long-range harmonic call used for territorial advertising and social cohesion [1,17–19]. Wolf howl acoustic structure has been shown to vary across individuals [1,20–24], groups [25], and subspecies [8,26]. Among the *Canis* clade, smaller species generally have howls that end in a sharp drop in frequency and a greater diversity of howl type usages [8]. Previous research has shown that Indian wolf howls generally have a higher mean fundamental frequency compared to other wolf subspecies, which may be attributed to its smaller body size [26]. Using a larger set of howls that are statistically classified by their acoustic features can provide a more robust description of the characteristics and diversity of Indian wolf howl types.

Along with the howl, wolves also communicate using seven to twelve other harmonic calls [27–29]. Harmonic calls are produced by the vibration of vocal folds in the larynx, which results in a series of multiple integral frequencies of the fundamental frequency [30]. Many of these other harmonic vocalisations are short-ranged, and due to difficulties in recording these calls, remain less studied compared to the wolf howl [31]. These short-ranged calls are essential for communicating passive or aggressive behaviour among social canids [31–33]. Grey wolves also use non-pitched or noisy calls, which are produced by the acoustic resonance of the vocal tract [19,34–36]. Instead of a specific frequency band, noisy calls possess concentrated acoustic energy around a particular frequency range. Therefore noisy calls do not have a clear pitch or distinct frequency band in their spectrograms [30].

The whimper, whine and yelp are various harmonic calls for communicating passive and friendly behaviour among wolves [19,32], whereas noisy calls such as growl and bark indicate varying levels of aggression [19,32]. The whimper, and whine vocalisations are similar to a crying sound with the whimper having a comparatively shorter duration than whine [19,34]. The whine vocalisation is mostly used for submissive behaviour, whereas the whimper is primarily used for greeting [19]. The yelp is a short and sharp cry vocalisation that is associated with submissive behaviour involving body contacts [19,34]. To communicate different levels of aggression behaviours, wolves use noisy calls, which consist of the growl, woof, and bark. Growl is a non-harmonic sound to show dominance in any interaction, whereas the woof vocalisation is a non-harmonic sound cue used by adults for their pups [19,34,37]. The bark is a short, low pitched sound with rapid frequency modulation and is used during aggressive defence [27,37], such as defending pups or defending a food resource [4,38]. Wolves also express communication through mixed vocalisation either by ‘successive emission’ or by ‘superimposition’ of two or more sound types [29]. A recent study on the Italian wolf (*Canis lupus italicus*) suggests six other types of calls may combine with howls to make a complex chorus vocalisation [39].

This study investigates the acoustic structure of harmonic vocalisations of Indian wolves and classifies these harmonic vocalisations using a statistical approach. We accumulated the vocalisation data from free-ranging and captive Indian wolves, which will be the first study to evaluate different types of vocalisations of this wolf subspecies. Using multivariate analyses, we describe and classify different harmonic calls to develop a vocal repertoire of the Indian wolf.

## Materials and methods

### Study species

The Indian wolf (*Canis lupus pallipes*) is among the smallest wolf subspecies with an average body weight of 20.75 kg [40]. Indian wolves are mostly found in grasslands and the edges of dense tropical deciduous forest on the Indian subcontinent [40–44]. The average home range of a pack varies from 180–250 km^2^ [40]. We recorded vocalisations from nine packs of free-ranging wolves and ten captive wolves from Jaipur Zoo. For captive wolves, we collected vocalisation data from 10 wolves: two adult pairs and six subadults. One adult male was recently captured from the wild near the city of Jaipur, Rajasthan, India. The rest of the Indian wolves are descendants of captive breeders at Jaipur Zoo.

### Study sites

**Fig 1:**
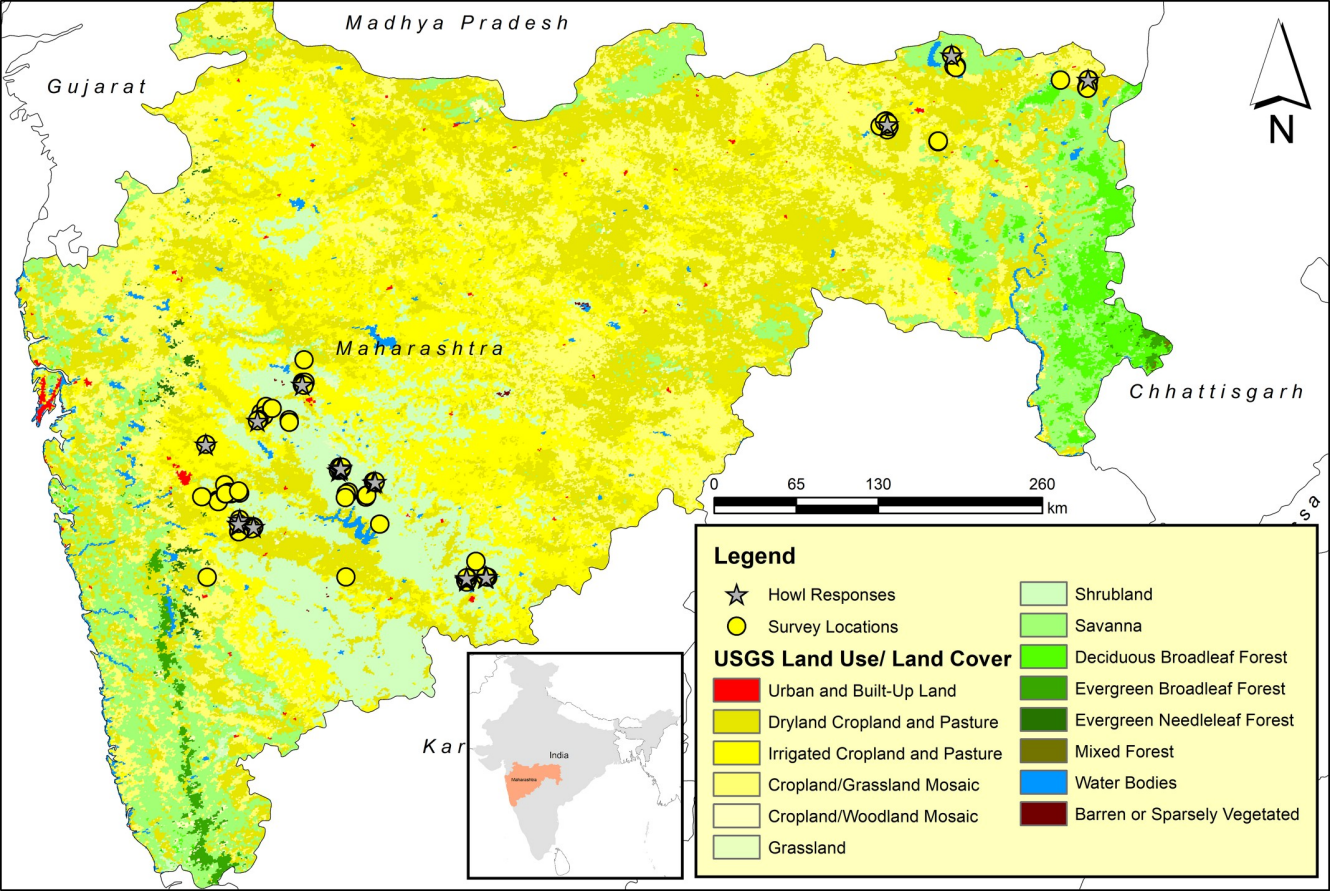
Map of survey sites of the free-ranging wolves. (Spatial Data Source: Political boundaries from Natural Earth, and Land Use/Land Cover data from USGS Earth Resources Observatory and Science (EROS) Center).

This study was conducted in the state of Maharashtra (Fig 1) and Jaipur Zoo of Jaipur, Rajasthan, India. The study site in Maharashtra was located on the central Deccan Plateau [45], which consists of the overlapping habitat of tropical dry deciduous forest, grassland, savanna (Western part) and tropical moist deciduous forest (Eastern part) [46].

### Data collection

Vocalisations of free-ranging wolves were recorded through acoustics survey from November 2015 to June 2016. The majority of the long-distance vocalisation recordings were collected through howling surveys to elicit howl behaviour. Opportunistically, spontaneous howls were also recorded. For other types of vocalisation data, we relied on opportunistic recordings from free-ranging wolves and captive wolves. Howl surveys were performed during early morning and evening hours using pre-recorded howls that were previously recorded from the Jaipur Zoo Indian wolves. Each howling session consisted of five trials with three minute long intervals [3]. A 50-second-long pre-recorded sequence of a solo howl was played three times using JBL Xtreme speakers (Harman Internation Industries, 2014) in order of increasing volume [3]. The session was followed by two 50-second-long chorus howls. In the case of a howling response, the session was terminated and repeated after 15 to 20 minutes [3]. Responses were recorded using Blue Yeti Pro Microphone (Blue Microphone, 2011) attached with Zoom H4N Handheld Audio Recorder (Zoom Corporation, 2009) at a sampling rate of 44.1KHz on 16-bit depth with 80 Hz noise filter. Along with howl surveys in the field, opportunistic recording sessions were conducted near wild Indian wolf den sites and rendezvous sites. In addition to howl surveys at Jaipur Zoo, vocalisations of captive wolves were recorded by installing microphones in the front of cages during closing hours (6:30 pm-7: 30 am).

### Ethical approval

The study on captive wolves in zoos was done with the permission of the Director of Jaipur Zoo and the Forest Department of Rajasthan, India [Letter no- 3(04)-II/CCFWL/2013/4586-87; Dated 30th Oct 2015]. The survey of free-ranging wolves of Maharashtra was performed with the consent of the Principal Chief Conservator of Maharashtra Forest Department [Letter no- 22(8)/WL/CR-947(14–15)/1052/2015-16; Dated- 6th Aug 2015]. No animal was harmed during the study, and the standard non-invasive protocol of howling survey was maintained.

### Feature extraction

We focused our analysis on harmonic vocalisations and excluded noisy calls since they do not possess a clear spectral band. Spectrograms of each vocalisation were generated through the Raven Pro 1.5 software [47] using the *Discrete Fourier Transform* (DFT) algorithm. The discrete Fourier function transforms the same length sequence of equally spaced sample points (N, where N is a prime number) with circular convolution being implemented on the points [48]. *Hann windows* were used at the rate of 1800 samples on 35.2 Hz 3dB filter. A total of 270 spectrograms were selected for further analysis based on clarity (i.e. clearer spectrogram with low noise and without external sound overlap). Web plot digitiser v3 [49] was used for digitising fundamental frequency from the spectral images. This digitised data was obtained at 0.1sec resolution. From this data, eleven acoustic variables (Table 1) were obtained based on their performance from previous studies [20,22].

**Table 1:** Acoustic variables based on fundamental frequency (f_0_) that were extracted for this study.

Variable Name	Definition of Variable
Min f	The minimum frequency of the fundamental (f_0_)
Max f	The maximum frequency of f_0_
Range f	Range of f_0_; f_0_ = Max f–Min f
Mean f	Mean frequency of f_0_ at 0.1 s interval over the duration
Duration	Duration of Howl measured at f_0_; Duration = t_end_- t_start_
Abrupt_0.025_	Number of abrupt changes in f_0_ more than 25Hz at single time step (0.1sec)
Abrupt_0.05_	Number of abrupt changes in f_0_ more than 50Hz at single time step (0.1sec)
Abrupt_0.1_	Number of abrupt changes in f_0_ more than 100Hz at single time step (0.1sec)
Stdv	Standard Deviation of f_0._
Co-fm	Coefficient of frequency modulation of f_0_ = Σ|f(t)–f (t+1)|/(n–1) Χ 100/Mean f_0_
Co-fv	Coefficient of frequency variation of f_0_ = (SD/mean) Χ 100

### Statistical analysis

#### Principal Component Analysis (PCA)

To obtain a smaller set of variables that explain most of the dataset’s variation, we used a principal component analysis (PCA), which is an unsupervised statistical approach that extracts linearly uncorrelated variables from a suite of potentially correlated variables [50]. To simplify the interpretation of factors, we performed varimax rotation using Kaiser normalisation [51]. From our dataset of 270 vocalisations, we used eight scalar variables that are related to spectral structure (Range f, Duration, Abrupt_0.025_, Abrupt_0.05_, Abrupt_0.1_, Stdv, Co-fm, Co-fv) for PCA analysis through the software SPSS (v22). The first principal component (PC1) and second principal component (PC2) were used in the subsequent clustering analyses.

#### Cluster analysis

To classify the recorded vocalisations from the Indian wolf, we used agglomerative hierarchical clustering through the R package AGglomerative NESting (AGNES)[52]. The agglomerative hierarchical clustering algorithm measures the dissimilarity between single and groups of observations using a “bottom-up” approach, thereby constructing clusters [53]. Agglomerative hierarchical clustering was performed using Euclidean distances with PC1 and PC2 from the 270-vocalisation data using eight scalar variables. Subsequently, s*ilhouette clustering* was combined with AGNES to validate the number of clusters in our vocalisation data. Silhouette clustering measures the similarity of observation within its cluster compared to other clusters [54]. The average silhouette value (0 represents poor fit, 1 depicts the highest fit) describes *the evaluation of clustering validity* [54]. Average Silhouette width (S_*i*_) was calculated for 14 different solutions (2 to 15 clusters). The “solution” that provided the best fit was selected upon the maximum average silhouette value. The dendrogram was plotted using ‘*Circlize Dendrogram*’ in the package ‘*Dendextend’* in program R [55].

#### Discriminant Function Analysis (DFA)

Discriminant function analysis (DFA) was performed using PC1 and PC2 as an independent variable under the program SPSS (v22) to cross-validate the obtained clusters from AGNES. Predicted clusters that were determined by the maximum silhouette value were then used as a grouping variable to evaluate within-group covariance in DFA analysis. From these clusters, we then used the box plot to show the overall pattern and distribution characteristics of different vocal clusters.

## Results

### Principal component analysis

Two principal components (PC1 and PC2) were generated from the eight simple scalar variables through PCA based on Kaiser-Guttman Rule (Eigenvalue >1) [56]. PC1 and PC2 together explained 70.6% variance. PC1 was based on the variances of six acoustic parameters (Abrupt_0.025_, Abrupt_0.1_, Abrupt_0.05_, Co-fv, Range f, Stdv) whereas PC2 is explained by the variances of five parameters (Abrupt_0.1_, Co-fm, Duration, Range f, Stdv) (Table 2).

**Table 2:** The loadings of PC1 and PC2. Communalities are the proportional factors by which the importance of each variable is explained.

Acoustics Parameters	PC1 Loading after varimax rotation	PC2 loading after varimax Rotation	Communalities
Abrupt_0.025_	.858	0	0.741
Abrupt_0.1_	.602	.415	0.535
Abrupt_0.05_	.825	0	0.732
Co-fm	0	.945	0.898
Co-fv	.862	0	0.750
Duration	0	-.382	0.231
Range f	.852	.392	0.880
Stdv	.759	.556	0.885
% of Variance	48.946	21.692	

### Cluster analysis

**Fig 2:**
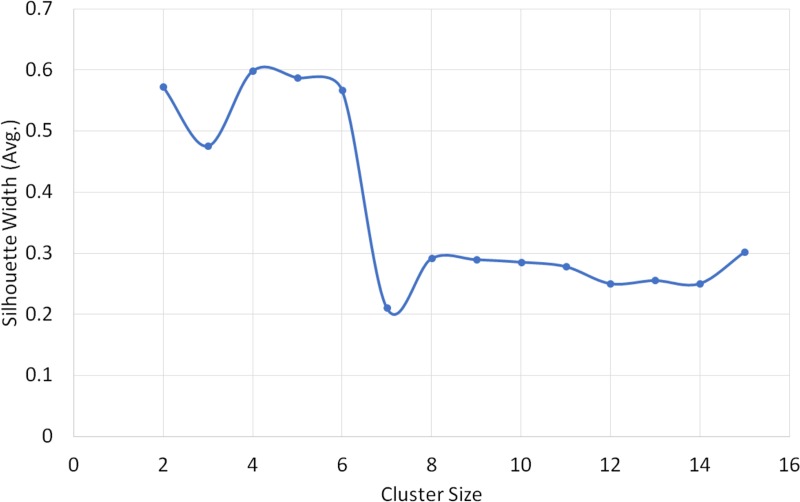
Average silhouette width plotted against 14 different solutions (2–15 cluster). Average Silhouette width represents the significance level (0 represents poor fit, 1 represents best fit). We obtained maximum silhouette width in 4 cluster solutions, i.e. S_i_ = 0.598.

**Fig 3:**
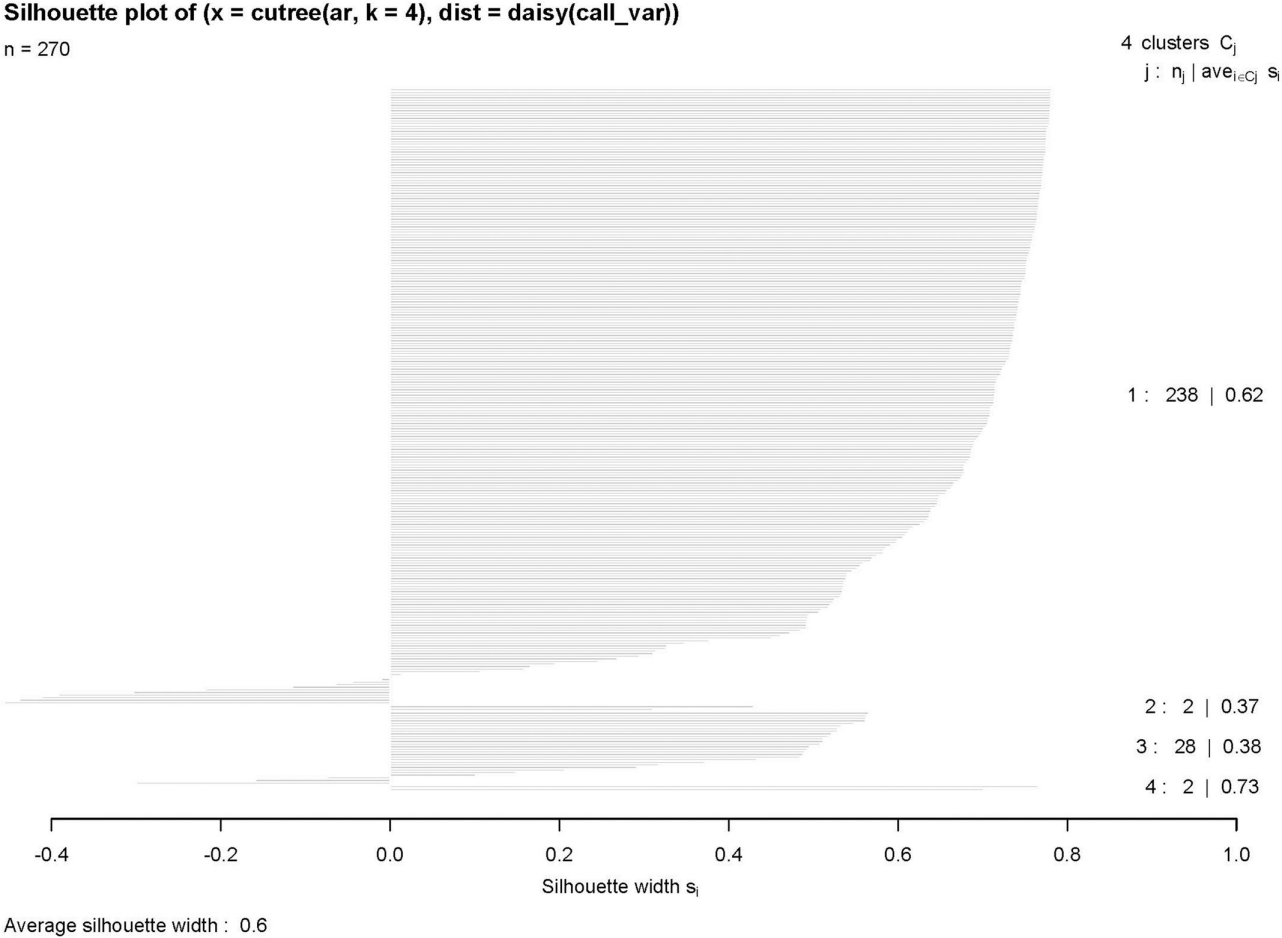
Silhouette plot showing the validation for the consistency among the clusters. This plot assesses the similarity or difference of each call from its clusters. A negative value indicates the chance of a call to fall under the neighbouring cluster. The average silhouette value of 4 groups are 0.62 (N = 238), 0.37 (N = 2), 0.38 (N = 28), 0.73 (N = 2) respectively.

**Fig 4:**
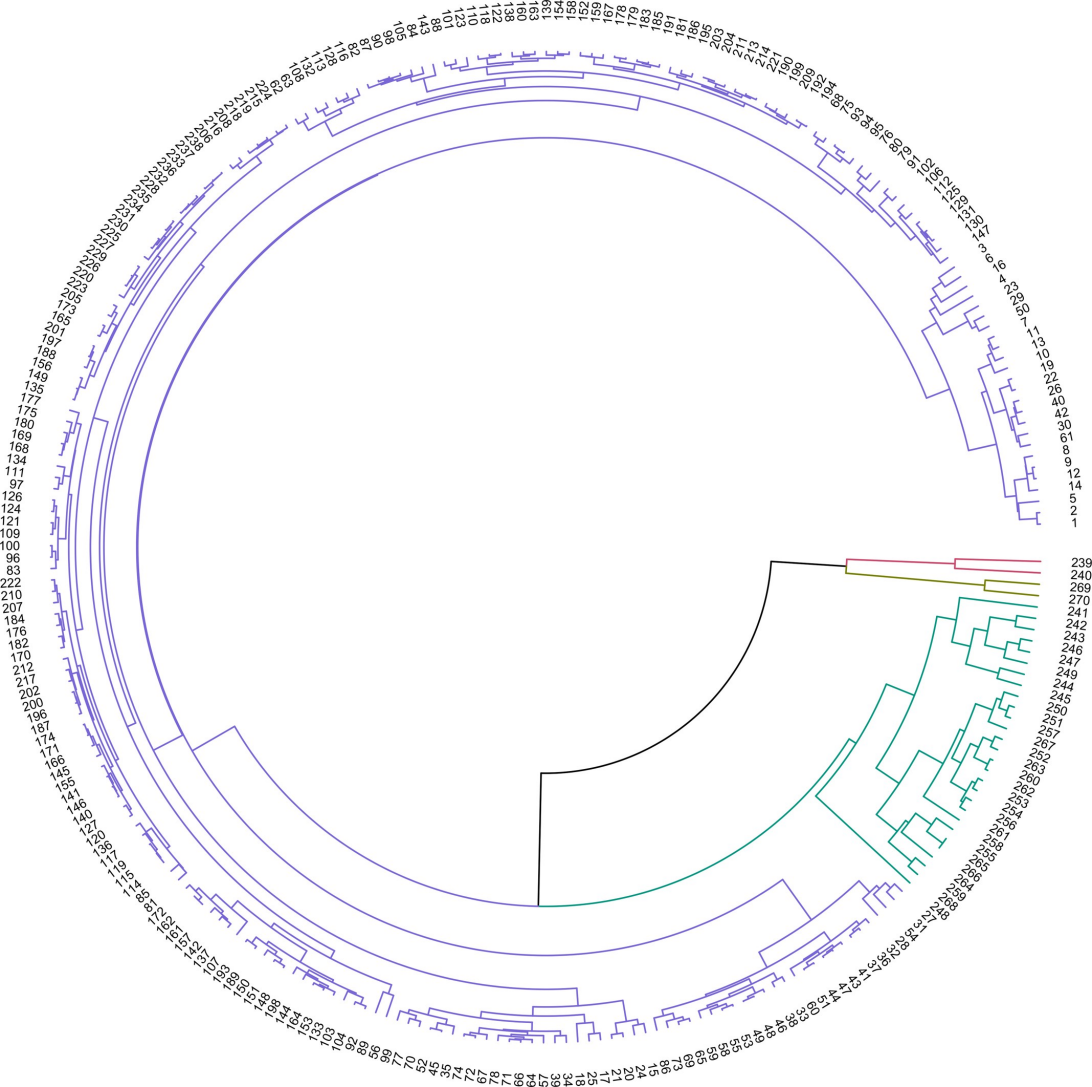
Cluster Diagram obtained from Agglomerative hierarchical clustering using Euclidean Distance as matric function. Four clusters were formed at 3.9 Clustering scale. Cluster 1 (Howl), Cluster 2 (Whimper), Cluster 3 (Social Squeak) and Cluster 4 (Whine) are denoted by the colours blue, red, green and brown, respectively.

The highest silhouette value (S_*i*_ = 0.598) was obtained at the 4-group solution in the cluster analysis using PC1 and PC2 from PCA analysis (Fig 2). The average silhouette value was 0.62 for the first cluster (N = 238), 0.37 for the second cluster (N = 2), 0.38 for the third cluster (N = 28) and 0.73 for the fourth cluster (N = 2) (Fig 3). The 4 clusters were formed at 3.9 clustering scale through agglomerative hierarchical clustering (Fig 4)

### Discriminant Function Analysis (DFA)

**Fig 5:**
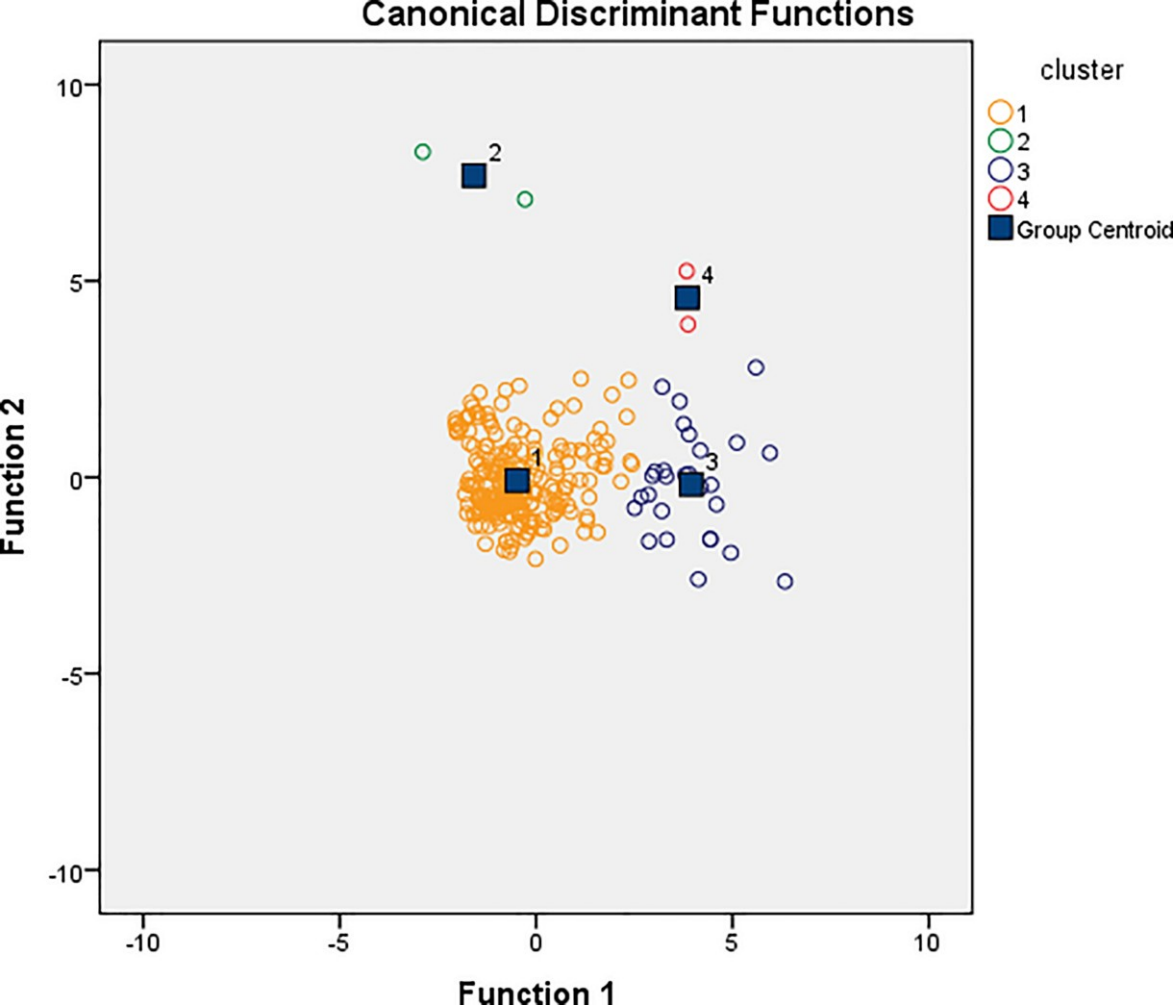
Plot for Discriminant Function Analysis (DFA) using PCA values for 270 vocalisation data from the Indian wolf. Different colours represent different call type.

DFA achieved 95.9% accuracy of vocal group identification using two PCA values (Table 3). Each of the four groups has a distinct group centroid. The graphical representation using two discriminant functions (DF1 and DF2) shows that vocal clusters do not overlap (Fig 5).

**Table 3:** Classification results of Discriminant Function Analysis (DFA). 95.9% of the vocal clusters (estimated from Agglomerative hierarchical clustering) are identified correctly.

		Cluster	Predicted Group Membership				Total
			1	2	3	4	
Predicted in Cluster analysis	Count	1	229	0	8	1	238
		2	0	2	0	0	2
		3	0	0	26	2	28
		4	0	0	0	2	2
	% Correct	1	96.2	.0	3.4	.4	100.0
		2	.0	100.0	.0	.0	100.0
		3	.0	.0	92.9	7.1	100.0
		4	.0	.0	.0	100.0	100.0

**Fig 6:**
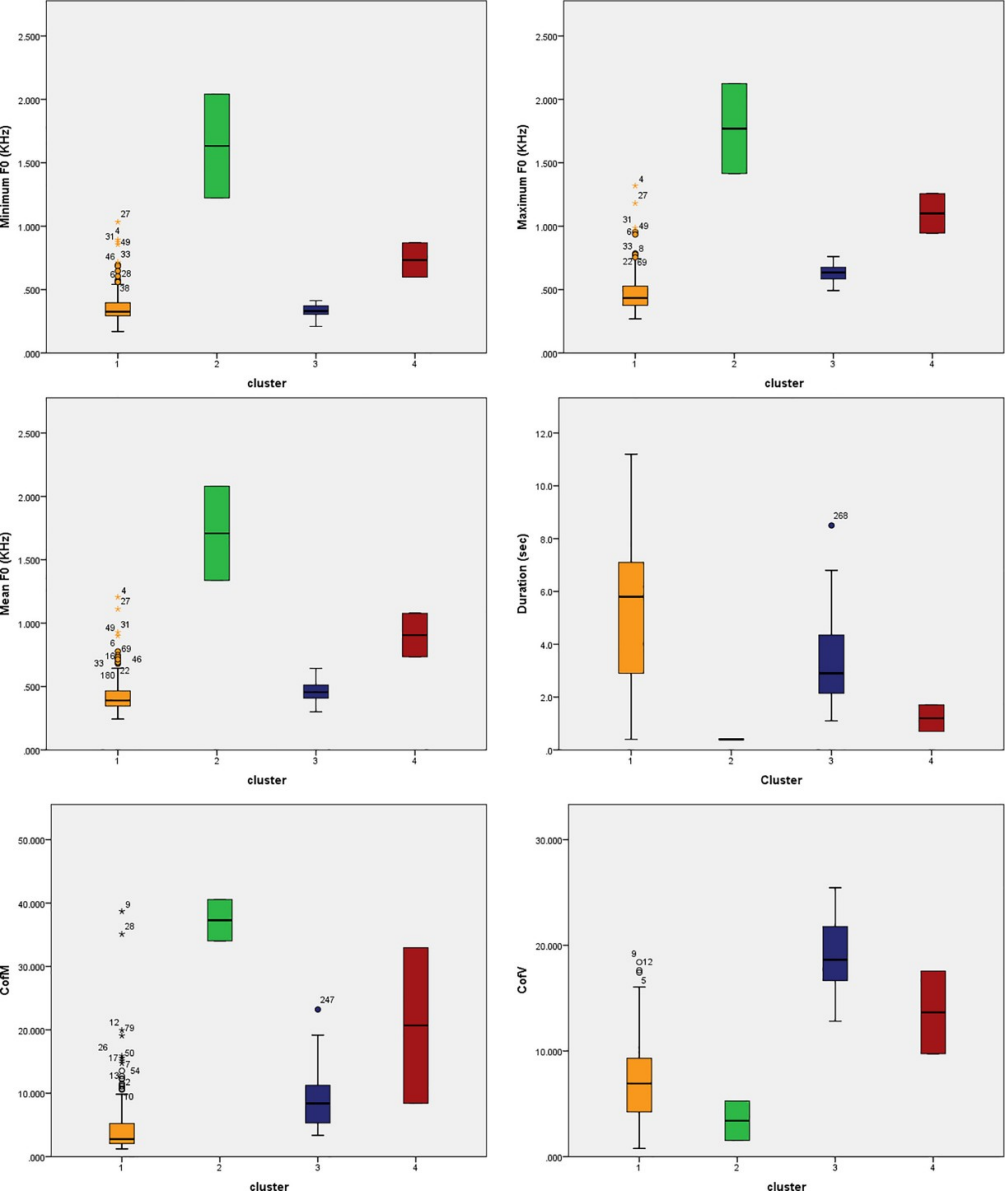
Box plot of variation among acoustic variables between different call type. a. Variation among minimum frequency, b. Variation among maximum frequency, c. Variation among Mean frequency, d. Variation among duration of the call, e. Variation among coefficient of frequency modulation, f. variation among coefficient of frequency variation.

The whisker box plot represents the variation among acoustic variables within the four identified call types (Fig 6). Call type 1 had the longest duration (5.214±2.49 Sec) whereas call type 2 showed the shortest duration among the four recognised groups (0.4±0) (N = 2). Type 2 calls also have high-frequency modulation (37.296±4.601) (variation in frequency per unit time). However, frequency variation (around the mean) is highest in type 3 calls (18.778±3.587) (Table 4).

**Table 4:** Variation among important acoustic variables within the four identified call types.

Cluster	Min f_0_ (Hz)	Max f_0_ (Hz)	Mean f_0_ (Hz)	Range f_0_ (Hz)	Duration (Sec)	Co-fv	Co-fm
1	359±116	469±141	422±126	110±65	5.21±2.49	7.17±3.689	4.444±4.463
2	1632±578	177±5	1708±524	137±77	0.4±0	3.407±2.632	37.296±4.601
3	327±51	623±77	461±83	295±50	3.47±1.85	18.778±3.587	9.071±4.802
4	733±190	1100±220	906±242	367±29	1.2±0.70	13.649±5.526	20.694±17.347

## Discussion

This study provides a quantitative assessment of the vocalisations of the Indian wolf subspecies. Our results show that there are four statistically classified groups of Indian wolf vocalisations based on ten captive individuals and nine free-ranging Indian wolf packs. Though the Four to Six solution groups showed a narrow difference in their average silhouette values based on *silhouette plot* analysis, the four cluster solution was found to be the most significant based on the global maxima. This characterisation of vocalisations provides a first step to evaluating the function and contextual use of different types of vocalisations in these canids.

**Fig 7:**
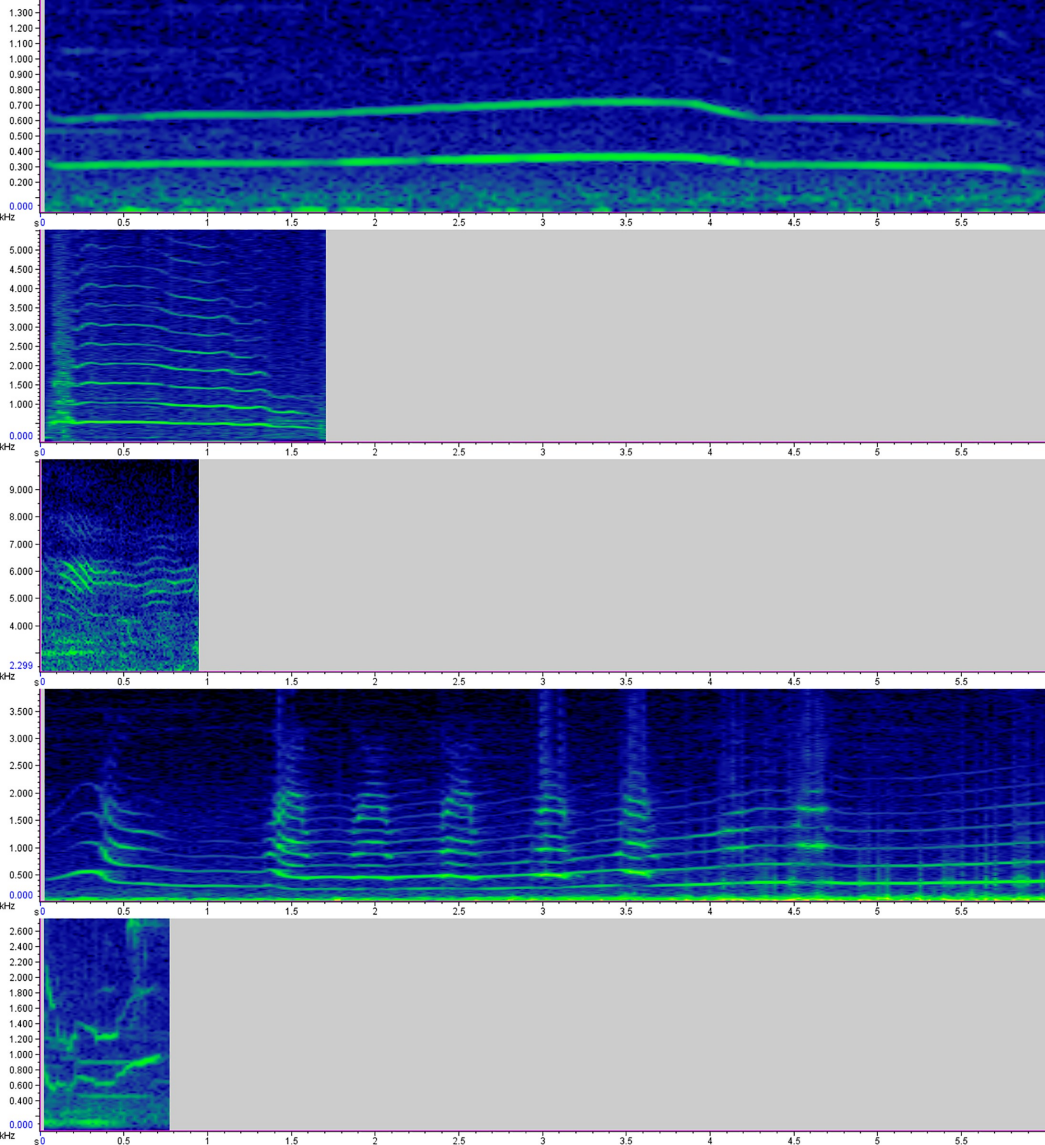
Spectrograms of different types of vocalisations of the Indian wolf. a. Howl (type 1); b. Bark-Howl (type 1 subtype); c. Whimper (type 2), d. Social Squeak (type 3); e. Whine (Type 4).

The first, most prolonged (5.214±2.49 sec) call type in our dataset is identified as a howl (Fig 7a). The fundamental frequency of the howl ranged from 359 Hz (±116) minimum to 469 Hz (±141) maximum (N = 238). Despite having a smaller body size, the mean fundamental frequency of the Indian wolf howl (422±126 Hz) was similar to the mean fundamental frequency of other wolf subspecies reported in previous studies [26]. This contrasts previous research that described Indian wolves as having a higher mean frequency in howls [26]. Our study’s large sample size of individuals and use of a classification model to statistically discriminate vocalizations may have aided in excluding other vocal types–such as howl barks–in our analyses to robustly describe Indian wolf howls. Additionally, variation in howl acoustic structure has been suggested to be partly individual-specific, which may be due to a combination of differences in body sizes, age class, or gross anatomy [1,20–24]. For example, the mean fundamental frequency of 11 Iberian wolf individuals was reported to range from 332Hz (±47) to 666Hz (±60) [21]. This high acoustic variation associated with individual wolves highlights the importance of having a large enough sample size of individual wolves to robustly describe vocal types and assess individual-specific variation within a population. To further understand the influence of body size on wolf howl acoustic structure, it would be important to identify howls using a classification-guided approach across all vocalization data of various subspecies as well as incorporating information of each howl’s associated wolf weight and individual’s identity.

Since the howl is the most detectable vocalisation used in long-range social cohesion and territorial advertisement [8,31], our high howl sample size shouldn’t be considered as the most frequent vocalisation. Barking-howl, which was mentioned by many authors as a common type of mix vocalisation in wolves [31,35,39], falls under the same cluster along with howling (Fig 7b). From our field observations, wolves bark in defence to an immediate threat. In one such occasion, the she-wolf of a pack started barking at nearby villagers to protect her pups and did not stop until all the three pups ran away to a safer distance from the villagers.

While the howl has been extensively studied in its behavioural function and variation across subspecies [8,26], less is known about short-range communication among wolves. Our study has identified and described three short-range communication call-types found in Indian wolves. Corresponding to our results, the second call type has the highest frequency modulation (37.296±4.601) and is commonly known as a whimper (Fig 7C). The whimper is low intensity but high-pitched sound that is used for short-distance communication among pack members [28,31] (mean fundamental frequency = 1708±524 Hz). This short duration (0.4±0 sec) vocalisation is reported to be associated with submissive or friendly greeting behaviour [31,35]. Since it is not audible from more than one to two hundred yards away [35], our dataset contains only a few observations (from different packs) of this type of call (N = 2). While our study provides some initial insight into the acoustic structure of this vocalisation, further sampling will be needed to characterize the acoustic structure of the whimper robustly.

The third group of Indian wolf vocalisations can be termed as *‘social squeak’* (Fig 7C), following observations by previous studies (Mech 1981, Crisler 1959, Fentress 1967). This high-frequency variable vocalisation (18.778±3.587) in the Indian wolf is similar to ‘talking’, which was defined as ‘hovering around one pitch’ [57]. The social squeak is considered to be context-dependent, with variation within the call type being dependent on differing social interactions among individuals [58]. Otherwise, there is little known about its function in wolf packs and if it’s a common communication across different canid species and within domestic dogs. Our results suggest that the social squeak has a minimum frequency of 327 Hz (±51) to Maximum of 623 Hz (±77) for Indian wolves (N = 28).

Lastly, our fourth vocal group we identified as the whine (Fig 7E), which is characterized as a short duration vocalisation (1.2±0.707 Sec). The whine is mainly used during stressful situations, such as pack separation and/or intra-pack conflict [30]. Additionally, female wolves have also been reported to whine during the nursing of pups in the den [59]. The whine in the Indian wolf (*Canis lupus pallipes*) is longer than the whine reported in Italian wolf (0.13±0.10 sec), which the Indian wolf has comparatively larger body size than Indian wolf [39]. Although our data set is too small (N = 2; from two different individuals) to interpret robustly, the mean fundamental frequency of Indian wolf whine (906±242 Hz) has a similar frequency as the Italian wolf (979±109 Hz) [39].

Arising from the challenges of monitoring elusive and low-density species, acoustic methods for detection and estimating population parameters has become increasingly utilized in wildlife management [60,61]. Early wolf biologists had recognized its effectiveness for detection [62], and further statistical work on howl acoustic structure has improved its ability to monitor wolf populations [63–65]. Statistically validating wolf howls from other vocalisations using an unsupervised classification technique avoids having a human biased sample of vocalisations for performing subsequent behavioural and statistical analyses, such as for identifying individuals [43]. It is important to note that howls can be context-dependent, in which individuals’ howl acoustic structure can vary according to certain behavioural contexts [24]. Since the howls were recorded from both elicited and spontaneous responses, our study’s characterization of the howl should be taken with caution, as it may comprise of multiple context-specific howls.

Further research on a larger dataset of Indian wolf vocalisations can develop a more robust classification of the vocal repertoire of this subspecies. Additionally, we defined call types in our study based on similarity to previously defined call types, such as whimper, whine, and social squeaks [35,66]. Incorporating information on the behaviour associated with these call types would aid in describing and validating the call types in our study. Therefore, statistical classification coupled with behavioural monitoring through a visual recorder is one future avenue of research, which will aid in decoding wolf behaviour in the context of its vocalisation. More broadly, the species within the *Canis* clade vary in their body sizes, social structure, and habitats [67]. The diversity of social complexity and vocal communication across species within *Canis* represents a unique system to address questions on the relationship between vocal communication and social complexity [68–70]. Therefore, describing the vocal repertoires of various canid taxa provides a first step into understanding the ecological, social, and phylogenetic factors influencing the diversity of vocal communication within the genus *Canis*.

## Supporting information

S1 File The variables, clusters and other details information of every calls. (PDF)
