# Supplementary material for: Characterising the harmonic vocal repertoire of the Indian wolf (Canis lupus pallipes)
Source: PLoS One. 2019 Oct 31;14(10):e0216186. doi: 10.1371/journal.pone.0216186 (PMC6822943; doi:10.1371/journal.pone.0216186)
Supplement: S1 File — (PDF) [file pone.0216186.s001.pdf]

Serial	cluster	neighbor	sil_width	RowLabels	MinimumF	Maximum	Range	Mean	duration
1	1	3	-0.45623	151108-00	0.383503	0.622051	0.238548	0.51086	2.3
2	1	3	-0.4375	151219-00	0.379763	0.620647	0.240884	0.515481	1.4
3	1	4	-0.41143	5a.wav etc	0.382079	0.707351	0.325272	0.643841	3
4	1	3	-0.39182	160428-00	0.892267	1.318505	0.426238	1.205132	5
5	1	3	-0.30294	151103-00	0.333526	0.625801	0.292275	0.468517	4.7
6	1	3	-0.21812	Waghapur_	0.686712	0.954899	0.268187	0.77748	2.9
7	1	3	-0.11596	151107-00	0.441516	0.643936	0.20242	0.554657	1.1
8	1	3	-0.06488	160130-00	0.445252	0.772789	0.327537	0.556224	4.2
9	1	3	-0.04466	151107-00	0.235215	0.388397	0.153182	0.334607	0.4
10	1	3	-0.01077	151104-00	0.439434	0.678573	0.239139	0.555277	1.4
11	1	3	0.002758	160608-00	0.473618	0.72341	0.249793	0.582389	3
12	1	3	0.014087	151106__2	0.261592	0.41263	0.151038	0.354882	0.7
13	1	3	0.106646	151104-00	0.440188	0.676	0.235812	0.555739	1.4
14	1	3	0.159222	4CH003M_	0.428408	0.603215	0.174808	0.503161	2
15	1	3	0.166224	151219-00	0.324414	0.545085	0.220671	0.423632	7.1
16	1	3	0.194879	5a.wav etc	0.400439	0.711503	0.311064	0.67976	4.1
17	1	3	0.24634	151219-00	0.3213	0.472949	0.15165	0.368993	1.4
18	1	3	0.268296	151220-00	0.341672	0.536366	0.194694	0.454424	5.2
19	1	3	0.294942	160130-00	0.403844	0.664971	0.261127	0.574589	4.6
20	1	3	0.31099	151219-00	0.310399	0.529088	0.218689	0.422934	6
21	1	3	0.314112	151108-00	0.240887	0.434103	0.193216	0.287968	3.9
22	1	3	0.32584	160130-00	0.529999	0.785329	0.25533	0.687774	4.2
23	1	3	0.326747	160608-00	0.437493	0.605379	0.167885	0.532603	2.7
24	1	3	0.347939	160203-00	0.280337	0.470602	0.190265	0.390485	6.4
25	1	3	0.376922	151219-00	0.324135	0.547898	0.223763	0.401823	4
26	1	3	0.450395	151108-00	0.414286	0.580906	0.16662	0.48517	1.1
27	1	2	0.46116	160307-00	1.032742	1.180682	0.14794	1.110612	1.4
28	1	2	0.472785	151106-00	0.561481	0.617808	0.056326	0.586791	0.4
29	1	3	0.485404	5a.wav etc	0.333944	0.527026	0.193082	0.377786	2.7
30	1	3	0.491991	151219-00	0.308526	0.460676	0.15215	0.392282	1.8
31	1	2	0.492069	160324-00	0.855396	0.986314	0.130918	0.927613	1
32	1	2	0.492297	160115-00	0.46396	0.47689	0.012931	0.469931	1.3
33	1	2	0.492975	4CH001M_	0.71575	0.776226	0.060476	0.747552	1.3
34	1	3	0.494555	151220-00	0.321271	0.561504	0.240232	0.417357	7.3
35	1	3	0.495034	151219-00	0.280638	0.458033	0.177395	0.38067	3.7
36	1	2	0.507089	160115-00	0.454364	0.467638	0.013273	0.462124	1.7
37	1	2	0.509311	160115-00	0.464461	0.48209	0.01763	0.474128	1.6
38	1	2	0.518055	151108-00	0.575363	0.631205	0.055842	0.605776	1.1
39	1	3	0.520329	160203-00	0.424085	0.616119	0.192034	0.512618	6.7
40	1	3	0.5241	160203-00	0.437704	0.611826	0.174122	0.544237	6.6
41	1	2	0.525293	160115-00	0.457989	0.472264	0.014275	0.466272	2.1
42	1	3	0.532253	160324-00	0.329431	0.504008	0.174576	0.436817	1.9
43	1	2	0.533165	4CH003M_	0.436383	0.452739	0.016357	0.443141	2.2
44	1	2	0.534372	160115-00	0.452459	0.474134	0.021675	0.467211	1.9
45	1	3	0.535124	160203-00	0.415425	0.591104	0.175678	0.555622	5.5
46	1	2	0.535963	4CH001M_	0.69461	0.744169	0.049558	0.717951	1.9
47	1	2	0.536184	160115-00	0.456646	0.472153	0.015507	0.465833	2.3
48	1	2	0.536969	4CH003M_	0.405677	0.438413	0.032736	0.429603	1.1
49	1	2	0.539056	151211-00	0.874199	0.934223	0.060024	0.89998	2.5

Serial	cluster	neighbor	sil_width	RowLabels	MinimumF	Maximum	Range	Mean	duration
50	1	2	0.539931	151108-00	0.541323	0.69829	0.156966	0.606406	0.9
51	1	2	0.539947	160115-00	0.444666	0.467638	0.022972	0.460151	1.9
52	1	3	0.54559	160203-00	0.419068	0.61956	0.200492	0.513602	6.2
53	1	2	0.55183	160324-00	0.448977	0.511399	0.062422	0.49062	1.1
54	1	2	0.555503	151106-00	0.569878	0.671564	0.101686	0.610487	0.9
55	1	2	0.5588	160324-00	0.430509	0.49268	0.062171	0.461859	1
56	1	3	0.568365	151220-00	0.336516	0.532171	0.195655	0.408089	9.8
57	1	3	0.569958	151107-00	0.251983	0.412902	0.160919	0.334575	4.5
58	1	2	0.5754	160324-00	0.428301	0.488807	0.060506	0.460158	1.2
59	1	2	0.582986	151108-00	0.559772	0.631666	0.071895	0.602515	1.2
60	1	2	0.583541	151108-00	0.293712	0.325918	0.032207	0.307695	1.6
61	1	3	0.58611	151219-00	0.278984	0.455629	0.176645	0.416823	3
62	1	3	0.591882	151219-00	0.298822	0.46506	0.166238	0.374198	2.2
63	1	3	0.597835	151219-00	0.4354	0.592748	0.157348	0.490246	3.8
64	1	3	0.599436	151108-00	0.218047	0.415285	0.197239	0.369992	6
65	1	2	0.605848	160324-00	0.444425	0.514327	0.069902	0.489725	1.1
66	1	3	0.609301	160203-00	0.394698	0.572259	0.177561	0.495666	6.3
67	1	3	0.611849	151106-00	0.203	0.386762	0.183762	0.354263	6.2
68	1	2	0.614346	160428-00	0.371429	0.402871	0.031442	0.389681	2.8
69	1	2	0.618146	5a.wav etc	0.684182	0.776814	0.092632	0.732386	2.4
70	1	3	0.626606	151220-00	0.326953	0.533521	0.206568	0.465027	6.5
71	1	3	0.631293	151211-00	0.168791	0.334039	0.165248	0.284997	4.7
72	1	3	0.636112	160428-00	0.338095	0.541648	0.203552	0.414656	7.9
73	1	2	0.636895	160608-00	0.44575	0.550169	0.104419	0.49399	2.3
74	1	3	0.637169	151105-00	0.262236	0.426621	0.164385	0.384638	7
75	1	2	0.638981	160324-00	0.44411	0.493741	0.049631	0.484187	3.1
76	1	2	0.63972	151108-00	0.437624	0.543563	0.105939	0.498124	1.1
77	1	3	0.644819	151220-00	0.341953	0.514521	0.172569	0.415375	5.4
78	1	3	0.646869	151108-00	0.218475	0.407497	0.189021	0.361987	5.9
79	1	2	0.647709	151106-00	0.457764	0.53084	0.073076	0.498745	0.9
80	1	2	0.648661	160608-00	0.470589	0.61829	0.147701	0.528986	2
81	1	2	0.649967	151104-00	0.313326	0.333577	0.020252	0.326905	6.8
82	1	2	0.656709	151107-00	0.307304	0.335705	0.028401	0.319858	6
83	1	2	0.658399	151104-00	0.329996	0.34826	0.018264	0.340328	8.2
84	1	2	0.662988	151104-00	0.315885	0.349226	0.033341	0.338123	5.6
85	1	2	0.665307	151105-00	0.341371	0.367691	0.026321	0.356657	7.6
86	1	2	0.666536	151219-00	0.396216	0.461947	0.065732	0.424002	1.3
87	1	2	0.672101	160203-00	0.277449	0.304668	0.027219	0.293098	5.8
88	1	2	0.674569	160203-00	0.476205	0.540549	0.064344	0.521478	4.8
89	1	3	0.676649	151105-00	0.225133	0.345196	0.120063	0.295537	9.4
90	1	2	0.678037	151107-00	0.281323	0.314985	0.033662	0.297658	5.9
91	1	2	0.678312	160428-00	0.468953	0.606053	0.137099	0.520182	2.3
92	1	3	0.678546	151105-00	0.195399	0.378456	0.183057	0.320078	7.9
93	1	2	0.678985	160203-00	0.328915	0.398959	0.070045	0.37024	2.9
94	1	2	0.680878	160428-00	0.367627	0.441009	0.073382	0.407829	3
95	1	2	0.684612	151105-00	0.278689	0.338633	0.059944	0.319483	3
96	1	2	0.685877	151105-00	0.328781	0.378421	0.04964	0.346519	8.5
97	1	2	0.685893	151103-00	0.326135	0.36088	0.034745	0.349691	9.1
98	1	2	0.68746	160428-00	0.277254	0.31663	0.039376	0.300507	5.9

Serial	cluster	neighbor	sil_width	RowLabels	MinimumF	Maximum	Range	Mean	duration
99	1	3	0.687657	151108-00	0.230061	0.418619	0.188558	0.374205	6.2
100	1	2	0.689517	151106-00	0.330396	0.356308	0.025913	0.348079	8.1
101	1	2	0.689811	151105-00	0.312216	0.355914	0.043698	0.325649	4
102	1	2	0.690548	160203-00	0.559097	0.666238	0.107141	0.616731	2.9
103	1	3	0.692738	151107-00	0.320333	0.463309	0.142976	0.398087	7.7
104	1	3	0.695529	151108-00	0.255784	0.402671	0.146887	0.349809	8
105	1	2	0.698769	160428-00	0.36721	0.410538	0.043327	0.396102	5.9
106	1	2	0.700298	160203-00	0.289477	0.404181	0.114704	0.350555	2
107	1	2	0.701434	151108-00	0.278151	0.328084	0.049933	0.311796	10.2
108	1	3	0.704369	160324-00	0.387532	0.50286	0.115328	0.437872	3.4
109	1	2	0.706392	151107-00	0.292933	0.328458	0.035525	0.3168	7.8
110	1	2	0.706783	151102-00	0.285585	0.34464	0.059054	0.335454	4.9
111	1	2	0.707147	151107-00	0.288259	0.333697	0.045439	0.320155	8.9
112	1	2	0.708864	160130-00	0.396605	0.532074	0.135468	0.489546	2.8
113	1	2	0.709356	160531-00	0.478802	0.619192	0.14039	0.556463	4.3
114	1	2	0.709884	151108-00	0.301282	0.335347	0.034066	0.318472	6.6
115	1	2	0.709884	151108-00	0.301282	0.335347	0.034066	0.318472	6.6
116	1	3	0.712135	151106-00	0.463776	0.573261	0.109484	0.500179	2.9
117	1	2	0.713704	151103-00	0.331688	0.374649	0.04296	0.356614	6.6
118	1	2	0.713827	160428-00	0.360087	0.425904	0.065817	0.402548	4.9
119	1	2	0.714463	151106-00	0.338304	0.401174	0.06287	0.361734	7.2
120	1	2	0.71491	151107-00	0.316068	0.358479	0.042411	0.343598	7
121	1	2	0.7152	151105-00	0.299434	0.339974	0.04054	0.320606	7.7
122	1	2	0.715405	160428-00	0.359519	0.425351	0.065832	0.402415	5
123	1	2	0.715406	151108-00	0.283773	0.347356	0.063583	0.326661	3.5
124	1	2	0.715558	Waghapur_	0.314461	0.367009	0.052549	0.342577	7.8
125	1	2	0.717016	160324-00	0.386105	0.500275	0.11417	0.440848	3.3
126	1	2	0.717037	151104-00	0.319414	0.358431	0.039016	0.343142	7.4
127	1	2	0.718361	151108-00	0.292895	0.337565	0.04467	0.318941	7.1
128	1	2	0.721788	151219-00	0.385691	0.504671	0.11898	0.443906	2.6
129	1	2	0.722522	160428-00	0.381413	0.463343	0.08193	0.439643	2.2
130	1	2	0.723851	151219-00	0.308967	0.393036	0.084068	0.33928	2.6
131	1	2	0.725203	151220-00	0.337773	0.422445	0.084672	0.392026	2.9
132	1	2	0.727478	151104-00	0.29394	0.451691	0.157751	0.395546	4.7
133	1	3	0.72839	151106-00	0.251163	0.393399	0.142236	0.36072	7.8
134	1	2	0.731161	151105-00	0.300558	0.350433	0.049875	0.330718	8.6
135	1	3	0.731831	151220-00	0.30207	0.468384	0.166314	0.406998	6.3
136	1	2	0.732373	151219-00	0.306162	0.364967	0.058805	0.339678	6.6
137	1	2	0.732412	151220-00	0.364888	0.460714	0.095826	0.417144	11.1
138	1	2	0.733517	160203-00	0.385467	0.466701	0.081235	0.421065	5
139	1	2	0.734342	160526-00	0.381404	0.46831	0.086907	0.432756	4.7
140	1	2	0.73471	151107-00	0.288762	0.339974	0.051212	0.327612	7
141	1	2	0.736198	151107-00	0.316074	0.369354	0.05328	0.350214	7.2
142	1	2	0.736237	151220-00	0.358188	0.466225	0.108037	0.41351	11.2
143	1	2	0.736706	160428-00	0.392893	0.447164	0.054271	0.427946	5.8
144	1	3	0.736886	151107-00	0.322497	0.440224	0.117727	0.383599	8.2
145	1	2	0.737266	151107-00	0.276342	0.364905	0.088563	0.316812	8.1
146	1	2	0.738205	151103-00	0.317851	0.372193	0.054342	0.34144	7
147	1	2	0.73847	151103-00	0.232021	0.299023	0.067002	0.272837	2.4

Serial	cluster	neighbor	sil_width	RowLabels	MinimumF	Maximum	Range	Mean	duration
148	1	3	0.738506	151107-00	0.321454	0.425455	0.104001	0.376158	8.6
149	1	3	0.74029	160428-00	0.329699	0.4905	0.160801	0.423078	6.5
150	1	3	0.740471	151106-00	0.262292	0.378401	0.116109	0.328856	8.9
151	1	3	0.740774	151107-00	0.32067	0.426452	0.105782	0.377438	8.5
152	1	2	0.741892	160203-00	0.396152	0.529877	0.133725	0.479319	4.6
153	1	3	0.742562	151220-00	0.339597	0.492639	0.153042	0.410239	8.6
154	1	2	0.742604	160428-00	0.354709	0.435456	0.080746	0.405844	3.9
155	1	2	0.742745	151108-00	0.321678	0.372357	0.050679	0.349579	7.1
156	1	3	0.743272	160130-00	0.399963	0.55319	0.153227	0.486722	6.9
157	1	2	0.744103	160428-00	0.349728	0.463894	0.114166	0.411191	10.8
158	1	2	0.744244	151211-00	0.415395	0.47876	0.063365	0.454621	3.5
159	1	2	0.745674	4CH001M_	0.309716	0.390337	0.080621	0.356802	2.4
160	1	2	0.745772	160428-00	0.335209	0.410483	0.075274	0.377002	4.8
161	1	2	0.746001	151105-00	0.253123	0.330288	0.077165	0.295359	9.2
162	1	2	0.746001	151105-00	0.253123	0.330288	0.077165	0.295359	9.2
163	1	2	0.746878	Waghapur_	0.370324	0.466867	0.096542	0.433385	5.2
164	1	3	0.747168	151103-00	0.199674	0.294712	0.095038	0.265459	6.2
165	1	3	0.750561	151104-00	0.3228	0.451003	0.128203	0.39075	6.5
166	1	2	0.750928	151103-00	0.319233	0.373567	0.054334	0.354118	7.3
167	1	2	0.751338	151107-00	0.206787	0.298338	0.091551	0.276189	2.6
168	1	2	0.751397	151108-00	0.349052	0.407169	0.058117	0.375596	7.8
169	1	2	0.752022	151107-00	0.312216	0.372653	0.060437	0.342432	7.5
170	1	2	0.752311	151106-00	0.309658	0.362234	0.052576	0.333305	6.3
171	1	2	0.752496	151108-00	0.307675	0.372261	0.064585	0.347585	7.3
172	1	2	0.752612	151105-00	0.26106	0.335347	0.074287	0.305117	8.7
173	1	3	0.753753	160203-00	0.41424	0.563914	0.149674	0.497054	7.7
174	1	2	0.754056	151105-00	0.324895	0.38898	0.064085	0.370225	7.3
175	1	2	0.755974	160526-00	0.384081	0.457284	0.073203	0.424446	8.5
176	1	2	0.756224	151107-00	0.30759	0.374264	0.066674	0.349971	6.2
177	1	2	0.757035	151108-00	0.224673	0.295315	0.070642	0.268043	7.8
178	1	2	0.757068	151108-00	0.28285	0.396633	0.113783	0.376742	4.3
179	1	2	0.758575	4CH003M_	0.392941	0.460513	0.067572	0.434704	3.4
180	1	2	0.759326	151106__2	0.605028	0.7193	0.114272	0.691165	9.9
181	1	2	0.759944	151107-00	0.298768	0.390862	0.092094	0.34958	3.8
182	1	2	0.760146	160428-00	0.366004	0.432757	0.066753	0.390435	6.9
183	1	2	0.761923	151220-00	0.328902	0.441263	0.112361	0.401251	5.1
184	1	2	0.762354	151105-00	0.250323	0.331333	0.08101	0.319035	6.4
185	1	2	0.762555	151220-00	0.324395	0.443482	0.119086	0.401142	5.1
186	1	2	0.764038	160130-00	0.397611	0.526654	0.129043	0.470617	5.4
187	1	2	0.76427	151106-00	0.298704	0.381469	0.082765	0.352339	7.1
188	1	2	0.764829	160428-00	0.396373	0.558545	0.162171	0.444694	6.5
189	1	3	0.764882	151105-00	0.270581	0.367731	0.09715	0.32388	7.9
190	1	2	0.765152	151106__2	0.646592	0.754128	0.107535	0.713776	7.5
191	1	2	0.765428	151105-00	0.264862	0.363105	0.098242	0.323845	4.3
192	1	2	0.766096	151105-00	0.240475	0.300712	0.060238	0.270897	3.6
193	1	2	0.766137	151108-00	0.281792	0.378964	0.097172	0.33927	8.2
194	1	2	0.766579	160601-00	0.322317	0.425058	0.102741	0.390758	5.3
195	1	2	0.767139	151106-00	0.287583	0.410703	0.12312	0.377353	5.1
196	1	2	0.767699	151107-00	0.30759	0.376983	0.069393	0.350905	7

Serial	cluster	neighbor	sil_width	RowLabels	MinimumF	Maximum	Range	Mean	duration
197	1	2	0.768057	151108-00	0.27661	0.431853	0.155243	0.405531	6.3
198	1	3	0.768187	151107-00	0.311001	0.417753	0.106752	0.36584	7.8
199	1	2	0.768651	151106-00	0.298673	0.394547	0.095873	0.372284	5.8
200	1	2	0.770327	151105-00	0.246092	0.354771	0.108679	0.324264	7.4
201	1	2	0.770371	160130-00	0.414595	0.535636	0.121041	0.486152	6.1
202	1	2	0.770551	151108-00	0.321469	0.395714	0.074246	0.363073	7.2
203	1	2	0.77119	160526-00	0.366623	0.481928	0.115305	0.42623	5.6
204	1	2	0.771275	151220-00	0.367934	0.497265	0.129331	0.465905	6.1
205	1	2	0.771549	151220-00	0.347084	0.517021	0.169937	0.454471	7.9
206	1	2	0.771807	151107-00	0.267952	0.359169	0.091218	0.307682	7.3
207	1	2	0.77204	151105-00	0.268831	0.358479	0.089647	0.336979	6.1
208	1	2	0.772117	151220-00	0.320643	0.45474	0.134096	0.407349	7.9
209	1	2	0.772752	151108-00	0.311042	0.416749	0.105708	0.394026	5.7
210	1	2	0.77294	151103-00	0.324468	0.39582	0.071351	0.372358	6.4
211	1	2	0.773085	151220-00	0.345866	0.434265	0.0884	0.399721	5.1
212	1	2	0.773116	151107-00	0.292926	0.373772	0.080846	0.344373	6.7
213	1	2	0.774597	151107-00	0.318026	0.419601	0.101575	0.372562	5.2
214	1	2	0.774919	151104-00	0.314235	0.403502	0.089267	0.359074	4.9
215	1	2	0.775174	151220-00	0.293475	0.387625	0.094151	0.353044	6.8
216	1	2	0.775296	151104-00	0.326182	0.411557	0.085375	0.372211	7.5
217	1	2	0.775507	151108-00	0.313617	0.392045	0.078428	0.354624	6.9
218	1	2	0.775539	151104-00	0.332924	0.462663	0.129739	0.353961	7.2
219	1	2	0.775764	151220-00	0.335753	0.45182	0.116067	0.404392	7.6
220	1	2	0.775938	151108-00	0.307395	0.408605	0.10121	0.386804	5.9
221	1	2	0.776054	151107-00	0.272069	0.375638	0.103569	0.339206	5.1
222	1	2	0.776095	151105-00	0.291801	0.386209	0.094407	0.352619	6.4
223	1	2	0.776788	Waghapur_	0.367812	0.483298	0.115486	0.437251	6.9
224	1	2	0.778122	151220-00	0.3458	0.451003	0.105203	0.403074	7.4
225	1	2	0.779215	151103-00	0.319928	0.409471	0.089542	0.379083	6.9
226	1	2	0.779486	151103-00	0.3149	0.417319	0.102419	0.36312	5.9
227	1	2	0.779628	151103-00	0.295979	0.385107	0.089129	0.345203	5.4
228	1	2	0.77989	151108-00	0.234051	0.350304	0.116253	0.313801	5.8
229	1	2	0.780001	151107-00	0.284244	0.38161	0.097366	0.35027	5.8
230	1	2	0.780094	151107-00	0.18076	0.269558	0.088798	0.244035	4.9
231	1	2	0.780179	151108-00	0.265176	0.391401	0.126225	0.353552	6.8
232	1	2	0.780574	151107-00	0.303684	0.404723	0.101039	0.360278	6
233	1	2	0.780928	151107-00	0.312975	0.398003	0.085028	0.360753	6.2
234	1	2	0.781033	151107-00	0.281284	0.372357	0.091074	0.338025	6.3
235	1	2	0.781097	151105-00	0.312379	0.417596	0.105217	0.38059	6.8
236	1	2	0.781107	151106-00	0.246898	0.359156	0.112258	0.319147	6
237	1	2	0.781126	160203-00	0.39059	0.515204	0.124615	0.461667	7.5
238	1	2	0.78132	151107-00	0.311677	0.41147	0.099793	0.37544	6.5
270	4	3	0.765523	160324-00	0.867995	1.256659	0.388664	1.077935	1.7
269	4	3	0.701276	160531-00	0.59843	0.94518	0.34675	0.734388	0.7
268	3	4	0.565403	151108-00	0.209524	0.511117	0.301593	0.30831	8.5
267	3	4	0.563013	151219-00	0.346562	0.589469	0.242906	0.446605	3
265	3	4	0.562058	151105__1	0.260235	0.495777	0.235542	0.350381	2.9
266	3	4	0.562058	151105__1	0.260235	0.495777	0.235542	0.350381	2.9
264	3	4	0.54792	151219-00	0.372962	0.63394	0.260978	0.470654	6.8

Serial	cluster	neighbor	sil_width	RowLabels	MinimumF	Maximum	Range	Mean	duration
263	3	4	0.533061	151105__1	0.31755	0.521772	0.204222	0.394344	2.9
262	3	4	0.529217	151104-00	0.35024	0.619159	0.268919	0.441745	1.1
261	3	4	0.52845	151108-00	0.413259	0.697619	0.28436	0.557617	2.6
260	3	4	0.521439	151105__1	0.325688	0.602994	0.277306	0.451495	1.1
259	3	1	0.517027	151219-00	0.306428	0.578379	0.27195	0.450563	6.4
258	3	4	0.511733	151105__1	0.229508	0.497873	0.268365	0.4037	1.3
257	3	4	0.511487	160203-00	0.375087	0.663953	0.288866	0.479989	4.4
256	3	4	0.508061	160307-00	0.386833	0.6476	0.260767	0.517187	1.9
255	3	4	0.495783	151219-00	0.306072	0.669219	0.363147	0.414604	5.1
254	3	4	0.493794	151105__1	0.312779	0.593035	0.280256	0.433967	1.6
253	3	4	0.490052	151106__2	0.239535	0.49245	0.252915	0.300585	2.3
252	3	4	0.488142	151219-00	0.347352	0.635568	0.288216	0.49649	2.9
251	3	4	0.484847	160601-00	0.370534	0.63679	0.266257	0.476463	4.3
250	3	1	0.43306	151219-00	0.379733	0.625132	0.245399	0.460169	4.2
240	2	1	0.429452	151102-00	2.041102	2.12412	0.083018	2.079858	0.4
249	3	4	0.371585	160307-00	0.372768	0.713113	0.340344	0.527482	2
248	3	4	0.317757	151219-00	0.321641	0.675714	0.354073	0.441156	5.3
239	2	1	0.310573	151105__2	1.223398	1.416049	0.19265	1.337534	0.4
247	3	4	0.291178	160531-00	0.387444	0.70507	0.317626	0.564538	4.3
246	3	4	0.206237	160531-00	0.370463	0.749055	0.378592	0.642872	6
245	3	4	0.148824	151219-00	0.296313	0.612248	0.315935	0.372496	3.3
244	3	4	0.100941	151105__1	0.302609	0.678471	0.375862	0.503916	2.7
243	3	4	-0.07389	5a.wav etc	0.335999	0.674815	0.338816	0.571913	3.1
242	3	4	-0.15901	5a.wav etc	0.319269	0.676155	0.356886	0.58966	2.8
241	3	4	-0.29976	151104-00	0.359198	0.760228	0.40103	0.506095	1.5

Serial	cluster	gt.025	gt.5	gt.1	Stdv	CofM	CofV	PosMin	PosMax
1	1	6	3	0	0.068873	9.667379	13.48176	0.347826	1
2	1	8	2	0	0.067691	10.68508	13.13168	1	0.357143
3	1	4	1	1	0.097021	5.591139	15.06902	0.033333	0.333333
4	1	9	1	0	0.09052	2.74929	7.511227	1	0.66
5	1	3	1	0	0.081617	5.164223	17.42026	0.06383	0.93617
6	1	8	1	1	0.071059	6.822631	9.139607	0.517241	0.034483
7	1	6	2	0	0.067656	13.53211	12.19776	1	0.363636
8	1	5	1	0	0.064343	4.096134	11.56779	1	0.02381
9	1	2	2	0	0.061588	38.69177	18.40598	1	0.25
10	1	4	1	0	0.079415	10.69552	14.30194	0.785714	0.142857
11	1	4	1	0	0.079723	5.434803	13.68887	0.966667	0.066667
12	1	2	2	0	0.062556	19.93555	17.62724	0.857143	0.142857
13	1	6	0	0	0.073526	11.51722	13.23033	0.857143	0.142857
14	1	6	3	0	0.04185	8.84072	8.317464	1	0.15
15	1	4	0	0	0.06601	2.90507	15.58197	0.014085	0.549296
16	1	2	1	1	0.063322	3.8701	9.31537	0.02439	0.195122
17	1	6	1	0	0.046966	15.54138	12.7282	0.714286	1
18	1	3	1	0	0.063498	3.269596	13.97337	0.884615	0.25
19	1	2	1	0	0.071119	3.225734	12.37743	0.978261	0.304348
20	1	5	0	0	0.054437	3.844737	12.87133	0.833333	0.616667
21	1	6	0	0	0.037894	6.181339	13.15907	0.717949	0.025641
22	1	5	0	0	0.072764	3.490906	10.57969	1	0.190476
23	1	3	1	1	0.056881	5.657919	10.67989	1	0.111111
24	1	5	0	0	0.051016	3.282376	13.0648	1	0.171875
25	1	3	1	0	0.048754	4.412094	12.13313	1	0.05
26	1	6	0	0	0.052495	15.89331	10.81984	0.636364	0.090909
27	1	3	1	0	0.040967	9.637272	3.688694	0.571429	0.071429
28	1	1	0	0	0.022005	35.09525	3.750086	1	0.25
29	1	2	1	1	0.033151	6.536788	8.775175	0.185185	0.037037
30	1	3	1	0	0.047142	9.088742	12.0174	1	0.277778
31	1	3	0	0	0.036031	12.31005	3.88428	1	0.1
32	1	0	0	0	0.004464	8.836747	0.95001	0.076923	0.461538
33	1	0	0	0	0.019332	9.500044	2.586101	0.076923	0.615385
34	1	2	0	0	0.052979	3.068268	12.69403	0.013699	0.657534
35	1	2	1	0	0.047763	4.782642	12.54722	0.918919	0.108108
36	1	0	0	0	0.003607	6.843301	0.780491	1	0.529412
37	1	0	0	0	0.004667	7.125593	0.98437	1	0.5625
38	1	0	0	0	0.018281	10.64742	3.017818	1	0.272727
39	1	4	1	0	0.045341	2.761268	8.845089	0.014925	0.223881
40	1	3	1	1	0.042537	2.629679	7.815887	0.015152	0.257576
41	1	0	0	0	0.003776	5.607557	0.809775	0.047619	0.333333
42	1	1	0	0	0.06287	6.981491	14.39286	0.894737	0.105263
43	1	0	0	0	0.003912	5.430075	0.882819	0.045455	0.863636
44	1	0	0	0	0.006028	5.857772	1.290272	1	0.526316
45	1	4	1	0	0.049511	2.798857	8.910884	0.018182	0.581818
46	1	0	0	0	0.015936	6.602557	2.219627	0.368421	0.736842
47	1	0	0	0	0.004132	5.00864	0.887068	0.043478	0.304348
48	1	0	0	0	0.010771	10.53167	2.507214	0.909091	0.181818
49	1	0	0	0	0.014638	4.736683	1.626431	0.12	0.64

Serial	cluster	gt.025	gt.5	gt.1	Stdv	CofM	CofV	PosMin	PosMax
50	1	4	1	0	0.047504	15.11423	7.833625	0.888889	0.111111
51	1	0	0	0	0.00674	6.354738	1.464727	1	0.263158
52	1	2	1	0	0.05305	2.704329	10.32903	0.016129	0.225806
53	1	0	0	0	0.017206	11.09671	3.507069	1	0.272727
54	1	1	0	0	0.036272	14.72811	5.941479	0.666667	0.111111
55	1	0	0	0	0.01775	12.68173	3.843077	1	0.1
56	1	1	0	0	0.050869	2.121943	12.46513	0.030612	0.602041
57	1	0	0	0	0.053704	3.677686	16.05155	1	0.133333
58	1	0	0	0	0.01796	9.656889	3.903009	1	0.083333
59	1	1	0	0	0.024165	9.846504	4.010742	1	0.25
60	1	0	0	0	0.00861	7.818786	2.798127	0.8125	0.0625
61	1	1	1	0	0.047158	5.465667	11.31364	0.033333	0.966667
62	1	4	0	0	0.040129	7.094152	10.72391	1	0.181818
63	1	4	1	0	0.04046	4.154115	8.252914	0.052632	0.236842
64	1	2	0	0	0.044	2.862549	11.89217	0.016667	0.616667
65	1	1	0	0	0.020759	11.33425	4.238943	1	0.454545
66	1	4	0	0	0.046814	2.825394	9.44459	1	0.15873
67	1	1	0	0	0.046193	2.764089	13.0393	0.016129	0.516129
68	1	0	0	0	0.010055	4.781455	2.580408	0.571429	0.928571
69	1	1	0	0	0.026404	5.221716	3.605261	1	0.25
70	1	1	0	0	0.054355	2.543869	11.68862	0.984615	0.307692
71	1	0	0	0	0.041859	4.015605	14.68752	0.021277	0.510638
72	1	0	0	0	0.052078	2.041349	12.55944	0.924051	0.012658
73	1	0	0	0	0.024344	6.224422	4.928063	1	0.043478
74	1	0	0	0	0.052516	2.396287	13.65326	0.042857	0.728571
75	1	1	0	0	0.008488	3.660669	1.752983	1	0.580645
76	1	2	0	0	0.034277	12.22149	6.881148	1	0.454545
77	1	0	0	0	0.055673	3.465102	13.40298	0.296296	0.685185
78	1	1	0	0	0.043641	3.057265	12.05602	0.016949	0.644068
79	1	4	2	0	0.02404	19.06695	4.820184	0.888889	0.111111
80	1	2	0	0	0.037412	6.50557	7.072422	1	0.05
81	1	0	0	0	0.004171	1.669176	1.275928	1	0.044118
82	1	0	0	0	0.005251	1.953278	1.641523	1	0.016667
83	1	0	0	0	0.00489	1.398692	1.436974	0.97561	0.170732
84	1	0	0	0	0.006647	2.209487	1.965741	1	0.107143
85	1	0	0	0	0.005564	1.787454	1.560077	0.171053	0.013158
86	1	2	0	0	0.019166	11.07958	4.520233	0.769231	0.461538
87	1	0	0	0	0.006753	2.396626	2.303955	0.017241	0.568966
88	1	0	0	0	0.013781	2.639368	2.642649	0.020833	0.791667
89	1	2	0	0	0.026685	1.980117	9.029395	1	0.010638
90	1	0	0	0	0.00704	2.289318	2.365234	0.508475	0.016949
91	1	4	0	0	0.037258	8.088517	7.162403	0.521739	1
92	1	2	0	0	0.030068	1.691253	9.394029	1	0.012658
93	1	0	0	0	0.01719	5.41908	4.642894	0.034483	1
94	1	0	0	0	0.0194	4.639709	4.756918	0.033333	0.766667
95	1	0	0	0	0.014481	4.380557	4.532667	0.033333	0.9
96	1	0	0	0	0.006366	1.668544	1.837251	0.988235	0.011765
97	1	0	0	0	0.008374	1.363329	2.394661	0.010989	0.450549
98	1	0	0	0	0.007886	2.517026	2.624201	0.050847	0.271186

Serial	cluster	gt.025	gt.5	gt.1	Stdv	CofM	CofV	PosMin	PosMax
99	1	0	0	0	0.044483	2.747607	11.88746	0.016129	0.435484
100	1	0	0	0	0.009128	1.389033	2.622387	0.876543	0.333333
101	1	0	0	0	0.012554	3.348204	3.854927	0.125	0.95
102	1	4	1	0	0.034366	5.993858	5.572284	0.206897	0.37931
103	1	0	0	0	0.04833	2.096777	12.14048	0.012987	0.194805
104	1	0	0	0	0.04128	2.200094	11.80075	0.0875	0.4125
105	1	0	0	0	0.011914	2.299188	3.007852	0.050847	0.59322
106	1	0	0	0	0.031406	8.084416	8.958868	0.05	1
107	1	0	0	0	0.011517	1.60931	3.69379	0.990196	0.009804
108	1	4	0	0	0.037624	5.306729	8.592361	0.970588	0.029412
109	1	0	0	0	0.010162	1.540625	3.20773	0.025641	0.576923
110	1	0	0	0	0.012296	2.535958	3.665431	1	0.346939
111	1	0	0	0	0.010511	1.929506	3.283096	0.011236	0.685393
112	1	3	0	0	0.029149	5.21931	5.954268	1	0.071429
113	1	2	0	0	0.047996	3.978975	8.625245	0.27907	0.860465
114	1	0	0	0	0.010935	2.084778	3.433633	0.19697	0.651515
115	1	0	0	0	0.010935	2.084778	3.433633	0.19697	0.651515
116	1	5	0	0	0.035504	5.914827	7.098289	0.517241	1
117	1	0	0	0	0.012075	1.939266	3.385995	0.121212	0.393939
118	1	0	0	0	0.016009	2.43675	3.976884	1	0.122449
119	1	0	0	0	0.010311	1.809808	2.850328	0.986111	0.013889
120	1	0	0	0	0.011639	1.795809	3.387255	0.985714	0.4
121	1	0	0	0	0.011253	1.724389	3.510002	0.012987	0.350649
122	1	0	0	0	0.01595	2.380173	3.963579	1	0.14
123	1	0	0	0	0.017321	3.956476	5.302529	0.057143	0.6
124	1	0	0	0	0.010811	1.970068	3.155664	0.205128	0.012821
125	1	0	0	0	0.034515	3.911414	7.82917	0.969697	0.121212
126	1	0	0	0	0.012247	1.816043	3.569135	0.108108	0.756757
127	1	0	0	0	0.011251	1.883753	3.527613	0.985915	0.140845
128	1	5	0	0	0.026713	6.904339	6.017648	1	0.692308
129	1	3	0	0	0.022143	6.322462	5.036551	1	0.272727
130	1	1	0	0	0.02054	6.948422	6.054044	0.846154	0.076923
131	1	0	0	0	0.030616	4.849393	7.809754	0.034483	0.689655
132	1	0	0	0	0.043788	3.884487	11.07019	0.042553	0.595745
133	1	0	0	0	0.038926	1.940078	10.79118	0.038462	0.679487
134	1	0	0	0	0.015399	1.607722	4.656174	0.197674	0.546512
135	1	0	0	0	0.043553	2.561222	10.70104	0.015873	0.650794
136	1	0	0	0	0.013533	2.272768	3.983919	0.030303	0.636364
137	1	0	0	0	0.023553	1.432682	5.646309	0.009009	0.405405
138	1	0	0	0	0.020084	2.851299	4.769901	0.02	1
139	1	0	0	0	0.02243	3.017776	5.182961	0.021277	0.638298
140	1	0	0	0	0.013894	1.857748	4.241001	0.014286	0.414286
141	1	0	0	0	0.014821	1.810121	4.23194	0.027778	0.444444
142	1	0	0	0	0.026346	1.853056	6.37134	0.017857	0.410714
143	1	1	0	0	0.014647	3.293017	3.422607	0.155172	0.396552
144	1	0	0	0	0.040414	1.835219	10.53539	0.012195	0.341463
145	1	0	0	0	0.011073	1.655492	3.49513	1	0.012346
146	1	0	0	0	0.014827	1.89816	4.342616	0.085714	0.4
147	1	0	0	0	0.021579	5.880166	7.909287	1	0.375

Serial	cluster	gt.025	gt.5	gt.1	Stdv	CofM	CofV	PosMin	PosMax
148	1	0	0	0	0.038392	1.668016	10.20628	0.965116	0.44186
149	1	0	0	0	0.044028	2.520467	10.40663	1	0.323077
150	1	0	0	0	0.031055	2.395538	9.443416	0.011236	0.640449
151	1	0	0	0	0.03844	1.679228	10.18445	0.976471	0.341176
152	1	1	0	0	0.03847	3.355497	8.025923	0.021739	0.326087
153	1	0	0	0	0.039462	1.966338	9.619156	1	0.383721
154	1	1	0	0	0.02173	3.971025	5.354307	0.051282	0.948718
155	1	0	0	0	0.01646	1.840781	4.708453	0.028169	0.690141
156	1	0	0	0	0.0496	2.358156	10.19059	1	0.231884
157	1	0	0	0	0.02723	1.717282	6.622247	0.990741	0.018519
158	1	3	0	0	0.015221	4.794736	3.34812	0.171429	0.657143
159	1	3	0	0	0.024653	7.956452	6.90938	0.833333	0.208333
160	1	0	0	0	0.021147	3.379233	5.609168	1	0.145833
161	1	0	0	0	0.020911	2.203193	7.079994	0.5	0.086957
162	1	0	0	0	0.020911	2.203193	7.079994	0.5	0.086957
163	1	0	0	0	0.022989	2.712932	5.304504	0.019231	0.596154
164	1	0	0	0	0.030488	2.749929	11.48483	0.032258	0.774194
165	1	0	0	0	0.041947	2.438365	10.73496	0.061538	0.461538
166	1	0	0	0	0.01844	1.758376	5.207377	0.054795	0.739726
167	1	0	0	0	0.028174	4.711539	10.20114	1	0.346154
168	1	0	0	0	0.019684	1.738671	5.240808	0.948718	0.512821
169	1	0	0	0	0.017894	1.872112	5.225578	0.053333	0.64
170	1	0	0	0	0.017914	2.08029	5.374656	0.095238	0.809524
171	1	0	0	0	0.017524	1.986937	5.041747	0.013699	0.383562
172	1	0	0	0	0.025341	2.079251	8.305218	0.551724	0.16092
173	1	1	0	0	0.042166	1.869294	8.483086	1	0.038961
174	1	0	0	0	0.018896	1.784163	5.103821	0.054795	0.383562
175	1	0	0	0	0.022642	1.64898	5.334538	0.929412	0.258824
176	1	0	0	0	0.018762	2.253663	5.361006	0.048387	0.354839
177	1	0	0	0	0.016957	2.39799	6.326152	1	0.320513
178	1	1	0	0	0.022731	3.459707	6.033605	0.023256	0.55814
179	1	3	0	0	0.021795	4.911642	5.013805	0.794118	0.882353
180	1	0	0	0	0.027266	1.224548	3.944967	1	0.434343
181	1	0	0	0	0.033039	3.838548	9.451049	0.157895	0.657895
182	1	0	0	0	0.021257	2.19817	5.444476	0.26087	0.666667
183	1	0	0	0	0.026527	3.188264	6.610985	0.019608	0.803922
184	1	0	0	0	0.01752	1.870936	5.491628	1	0.359375
185	1	0	0	0	0.02727	2.954682	6.798029	0.019608	0.784314
186	1	1	0	0	0.033219	2.912393	7.058709	1	0.111111
187	1	1	0	0	0.015592	2.545609	4.425231	0.014085	0.690141
188	1	1	0	0	0.033739	2.502527	7.587029	1	0.015385
189	1	0	0	0	0.029802	2.091992	9.201428	0.253165	0.721519
190	1	0	0	0	0.035603	2.006825	4.988023	0.12	0.746667
191	1	0	0	0	0.024791	2.860207	7.65524	1	0.046512
192	1	1	0	0	0.018463	5.945842	6.815417	0.5	0.416667
193	1	0	0	0	0.028891	1.884261	8.515541	0.036585	0.695122
194	1	0	0	0	0.025839	3.158925	6.612551	0.037736	0.962264
195	1	0	0	0	0.03269	3.472668	8.663069	0.019608	0.647059
196	1	0	0	0	0.021919	1.93553	6.246288	0.057143	0.4

Serial	cluster	gt.025	gt.5	gt.1	Stdv	CofM	CofV	PosMin	PosMax
197	1	1	0	0	0.031055	2.395695	7.65795	0.015873	0.539683
198	1	0	0	0	0.034037	1.967971	9.303841	0.051282	0.346154
199	1	0	0	0	0.022912	2.773777	6.154403	0.017241	0.948276
200	1	0	0	0	0.018036	1.627087	5.562059	1	0.013514
201	1	2	0	0	0.033705	2.86614	6.933038	0.983607	0.311475
202	1	0	0	0	0.023597	2.091644	6.499143	0.069444	0.416667
203	1	0	0	0	0.031541	2.840327	7.400015	1	0.196429
204	1	0	0	0	0.032299	2.44999	6.932495	1	0.57377
205	1	0	0	0	0.036563	2.276467	8.045169	0.025316	0.253165
206	1	1	0	0	0.018086	2.24304	5.878225	0.150685	0.013699
207	1	0	0	0	0.021289	2.367113	6.317583	0.016393	0.590164
208	1	0	0	0	0.035625	1.926178	8.745464	0.012658	0.582278
209	1	0	0	0	0.026732	2.457436	6.784238	0.017544	0.508772
210	1	0	0	0	0.024779	2.03784	6.654731	0.046875	0.640625
211	1	0	0	0	0.032555	3.130809	8.144321	0.019608	0.529412
212	1	0	0	0	0.022474	2.028405	6.526184	0.014925	0.447761
213	1	0	0	0	0.030504	2.777582	8.187656	0.057692	0.307692
214	1	0	0	0	0.031955	3.233065	8.899244	0.142857	0.795918
215	1	1	1	0	0.02254	2.214043	6.384452	1	0.735294
216	1	0	0	0	0.030182	1.906129	8.108772	0.066667	0.32
217	1	0	0	0	0.025085	2.026235	7.073696	0.057971	0.492754
218	1	2	0	0	0.02167	2.069345	6.122141	0.986111	0.013889
219	1	0	0	0	0.034911	1.900247	8.63305	0.013158	0.513158
220	1	0	0	0	0.026877	2.355657	6.948409	0.016949	0.355932
221	1	0	0	0	0.028739	2.75137	8.472359	1	0.490196
222	1	0	0	0	0.023329	2.505557	6.615974	0.015625	1
223	1	1	0	0	0.033472	2.268141	7.655027	0.014493	0.333333
224	1	0	0	0	0.033878	2.047651	8.404934	0.013514	0.743243
225	1	0	0	0	0.027946	2.040944	7.371914	0.072464	0.42029
226	1	0	0	0	0.027503	2.552839	7.574171	0.050847	0.40678
227	1	0	0	0	0.028896	2.592646	8.370605	0.018519	0.611111
228	1	0	0	0	0.026299	2.575962	8.380719	0.965517	0.017241
229	1	0	0	0	0.02721	2.439682	7.768205	0.034483	0.482759
230	1	1	0	0	0.020387	3.329948	8.354274	1	0.673469
231	1	0	0	0	0.028377	2.728959	8.026325	0.014706	0.367647
232	1	0	0	0	0.030191	2.41591	8.379787	0.016667	0.366667
233	1	0	0	0	0.028598	2.521283	7.927352	0.048387	0.33871
234	1	0	0	0	0.027857	2.423207	8.24097	0.031746	0.365079
235	1	0	0	0	0.030168	2.240488	7.926586	0.044118	0.367647
236	1	0	0	0	0.025409	2.249877	7.96142	1	0.583333
237	1	0	0	0	0.030965	1.808709	6.70722	1	0.173333
238	1	0	0	0	0.02913	2.615847	7.758906	0.138462	0.461538
270	4	6	6	2	0.105017	8.428003	9.742375	1	0.411765
269	4	6	4	1	0.128941	32.96134	17.55761	0.142857	1
268	3	5	2	1	0.0602	3.903251	19.52568	0.223529	0.011765
267	3	10	3	0	0.063353	8.29809	14.18544	0.7	0.1
265	3	18	1	1	0.059076	10.39974	16.86058	0.310345	0.034483
266	3	18	1	1	0.059076	10.39974	16.86058	0.310345	0.034483
264	3	14	4	0	0.060312	5.008174	12.81452	0.720588	0.014706

Serial	cluster	gt.025	gt.5	gt.1	Stdv	CofM	CofV	PosMin	PosMax
263	3	11	6	1	0.052355	11.49757	13.27638	0.758621	0.655172
262	3	7	4	0	0.086362	23.21845	19.55017	0.454545	1
261	3	9	2	0	0.093689	7.442061	16.80171	0.038462	0.769231
260	3	6	3	0	0.103821	16.20936	22.99496	1	0.272727
259	3	7	0	0	0.090455	3.536469	20.07605	1	0.484375
258	3	8	5	0	0.088389	14.49907	21.89466	1	0.076923
257	3	5	1	0	0.095999	4.520279	20.00025	0.727273	0.113636
256	3	8	4	0	0.074072	10.03264	14.32217	0.842105	0.052632
255	3	10	3	0	0.096747	5.424453	23.33488	1	0.215686
254	3	7	2	0	0.075783	11.43331	17.46282	0.9375	0.125
253	3	3	1	1	0.072741	11.64308	24.19984	0.043478	1
252	3	15	2	1	0.082088	8.503836	16.53361	1	0.103448
251	3	6	2	0	0.077115	5.197311	16.18484	0.023256	0.813953
250	3	8	2	0	0.061413	5.494625	13.34571	0.97619	0.119048
240	2	2	0	0	0.032154	34.0427	1.545978	1	0.25
249	3	14	1	1	0.088557	11.07153	16.78858	0.05	0.65
248	3	16	7	0	0.079033	6.724948	17.91502	0.018868	0.169811
239	2	2	2	1	0.070478	40.55023	5.269221	0.25	1
247	3	6	3	1	0.10928	4.637809	19.35752	0.023256	0.255814
246	3	5	3	1	0.112843	3.364792	17.55301	0.016667	0.6
245	3	7	4	2	0.090681	9.69717	24.34426	0.727273	0.090909
244	3	10	6	1	0.111851	10.08244	22.19632	0.074074	0.555556
243	3	4	2	1	0.123896	6.019886	21.66349	0.032258	0.870968
242	3	4	1	1	0.119646	6.57961	20.29074	0.035714	0.642857
241	3	7	4	2	0.128853	19.16334	25.4602	0.333333	1

Serial	cluster	EndF	FAC1_2	FAC2_2
1	1	0.622051	1.270106	-0.03624
2	1	0.379763	1.251337	-0.01839
3	1	0.618617	1.334286	0.510044
4	1	0.892267	1.262808	0.272349
5	1	0.587697	1.099345	-0.14718
6	1	0.865262	1.094888	0.419508
7	1	0.441516	0.966526	0.119087
8	1	0.445252	0.920506	0.009229
9	1	0.235215	0.903087	-0.04078
10	1	0.465583	0.89133	0.200311
11	1	0.520396	0.877336	0.092325
12	1	0.26752	0.86312	-0.04176
13	1	0.502586	0.800813	0.13878
14	1	0.428408	0.756745	-0.00796
15	1	0.332976	0.720323	-0.47052
16	1	0.633931	0.697104	0.532992
17	1	0.472949	0.678879	-0.13224
18	1	0.343658	0.652413	-0.24169
19	1	0.404697	0.634713	0.05137
20	1	0.312485	0.598273	-0.36907
21	1	0.242774	0.591918	-0.39227
22	1	0.529999	0.602284	0.07022
23	1	0.437493	0.567405	0.359932
24	1	0.280337	0.543468	-0.46488
25	1	0.324135	0.550556	-0.12619
26	1	0.509381	0.456446	0.072809
27	1	1.138114	-0.1395	0.504189
28	1	0.561481	-0.68348	0.471677
29	1	0.371916	0.343643	0.346542
30	1	0.308526	0.408252	-0.00501
31	1	0.855396	-0.32719	0.479128
32	1	0.465569	-1.03399	0.307794
33	1	0.718695	-0.80878	0.411053
34	1	0.321365	0.387274	-0.3305
35	1	0.291717	0.407722	-0.12757
36	1	0.454364	-1.04569	0.271521
37	1	0.464461	-1.02214	0.28286
38	1	0.575363	-0.79071	0.380109
39	1	0.440264	0.358277	-0.2884
40	1	0.440246	0.353111	0.06813
41	1	0.460877	-1.04184	0.235864
42	1	0.329445	0.334103	0.102913
43	1	0.441131	-1.03298	0.225082
44	1	0.452459	-0.99171	0.252334
45	1	0.44141	0.349605	-0.17447
46	1	0.72242	-0.85765	0.324425
47	1	0.457708	-1.0339	0.217413
48	1	0.409524	-0.88356	0.310326
49	1	0.888594	-0.88038	0.30819

Serial	cluster	EndF	FAC1_2	FAC2_2
50	1	0.541327	0.242037	0.285218
51	1	0.444666	-0.97687	0.250621
52	1	0.440607	0.334252	-0.16978
53	1	0.448977	-0.74961	0.341352
54	1	0.584127	-0.39858	0.394446
55	1	0.430509	-0.72852	0.33711
56	1	0.361752	0.198867	-0.54445
57	1	0.251983	0.289508	-0.24218
58	1	0.428301	-0.72566	0.310266
59	1	0.559772	-0.60369	0.331546
60	1	0.297131	-0.87273	0.225261
61	1	0.450889	0.256477	0.034289
62	1	0.298822	0.257091	-0.0443
63	1	0.454155	0.249185	-0.07784
64	1	0.378947	0.232491	-0.2857
65	1	0.444425	-0.60317	0.293749
66	1	0.394698	0.217352	-0.27253
67	1	0.312447	0.203625	-0.31301
68	1	0.40201	-0.88503	0.14408
69	1	0.684182	-0.58109	0.277184
70	1	0.336347	0.195999	-0.19042
71	1	0.226669	0.1757	-0.27217
72	1	0.338095	0.151582	-0.32896
73	1	0.44575	-0.56265	0.246771
74	1	0.423943	0.145839	-0.34023
75	1	0.44411	-0.8284	0.122999
76	1	0.437624	-0.25789	0.251131
77	1	0.409845	0.161921	-0.16024
78	1	0.37361	0.148089	-0.25156
79	1	0.505171	-0.01564	0.167434
80	1	0.470589	-0.15581	0.213687
81	1	0.313326	-1.01248	-0.1868
82	1	0.307304	-0.96992	-0.12197
83	1	0.330721	-1.00837	-0.30994
84	1	0.315885	-0.93591	-0.08926
85	1	0.36678	-0.98222	-0.25248
86	1	0.42271	-0.52098	0.196295
87	1	0.301778	-0.92784	-0.12832
88	1	0.505917	-0.81237	0.028752
89	1	0.225133	-0.14904	-0.62705
90	1	0.298654	-0.9115	-0.13185
91	1	0.606053	-0.0182	0.089134
92	1	0.195399	0.006429	-0.4363
93	1	0.398959	-0.66823	0.119706
94	1	0.402567	-0.64782	0.124633
95	1	0.305863	-0.7025	0.085122
96	1	0.333023	-0.92227	-0.31529
97	1	0.335776	-0.91229	-0.39351
98	1	0.285235	-0.88288	-0.13316

Serial	cluster	EndF	FAC1_2	FAC2_2
99	1	0.32627	0.062198	-0.21931
100	1	0.336466	-0.90996	-0.32315
101	1	0.344235	-0.78164	0.004649
102	1	0.653216	-0.02354	0.055014
103	1	0.321901	0.001256	-0.37399
104	1	0.332124	-0.03717	-0.42941
105	1	0.371655	-0.83925	-0.11863
106	1	0.404181	-0.28213	0.126223
107	1	0.285049	-0.80065	-0.51466
108	1	0.413448	0.026007	-0.10448
109	1	0.304732	-0.85348	-0.31078
110	1	0.285585	-0.76723	-0.05033
111	1	0.315529	-0.83274	-0.39534
112	1	0.396605	-0.196	0.081941
113	1	0.601898	-0.04621	0.004412
114	1	0.317211	-0.83702	-0.21591
115	1	0.317211	-0.83702	-0.21591
116	1	0.573261	-0.00163	-0.04759
117	1	0.340038	-0.81916	-0.1969
118	1	0.360087	-0.72358	-0.03211
119	1	0.342415	-0.82102	-0.21521
120	1	0.318917	-0.82274	-0.2347
121	1	0.308356	-0.82248	-0.30473
122	1	0.359519	-0.72479	-0.04039
123	1	0.307224	-0.64232	0.027922
124	1	0.350943	-0.8226	-0.28708
125	1	0.390022	-0.34024	0.091494
126	1	0.339366	-0.81756	-0.27631
127	1	0.292923	-0.81203	-0.25098
128	1	0.385691	-0.07678	-0.02336
129	1	0.381413	-0.37255	0.078771
130	1	0.333704	-0.47016	0.068478
131	1	0.419348	-0.40933	0.071244
132	1	0.373966	-0.04151	-0.08315
133	1	0.351215	-0.11209	-0.3836
134	1	0.315722	-0.72645	-0.39962
135	1	0.36398	-0.05178	-0.19565
136	1	0.317072	-0.74976	-0.20142
137	1	0.388965	-0.56153	-0.55893
138	1	0.466701	-0.63488	-0.03562
139	1	0.39493	-0.59126	-0.0083
140	1	0.307362	-0.74875	-0.25307
141	1	0.339974	-0.74273	-0.26081
142	1	0.386523	-0.48724	-0.57156
143	1	0.421192	-0.70803	-0.14262
144	1	0.333175	-0.16857	-0.41663
145	1	0.276342	-0.73537	-0.2948
146	1	0.330987	-0.73373	-0.24838
147	1	0.232021	-0.46665	0.02504

Serial	cluster	EndF	FAC1_2	FAC2_2
148	1	0.322859	-0.22161	-0.46145
149	1	0.329699	-0.07808	-0.1994
150	1	0.363835	-0.27053	-0.48945
151	1	0.322557	-0.21909	-0.4499
152	1	0.423801	-0.20443	-0.02575
153	1	0.339597	-0.16037	-0.37846
154	1	0.39321	-0.51586	-0.0028
155	1	0.343805	-0.71384	-0.26676
156	1	0.399963	-0.08618	-0.18892
157	1	0.357814	-0.45681	-0.53858
158	1	0.420271	-0.53287	-0.01587
159	1	0.320745	-0.2575	-0.01662
160	1	0.335209	-0.59298	-0.0586
161	1	0.260723	-0.51723	-0.50607
162	1	0.260723	-0.51723	-0.50607
163	1	0.417231	-0.56548	-0.04428
164	1	0.253764	-0.18572	-0.38679
165	1	0.337293	-0.12725	-0.26135
166	1	0.358102	-0.67159	-0.29104
167	1	0.206787	-0.26476	-0.03695
168	1	0.353447	-0.65966	-0.32237
169	1	0.334867	-0.66169	-0.30707
170	1	0.330966	-0.66419	-0.22012
171	1	0.348703	-0.66538	-0.27964
172	1	0.266325	-0.43431	-0.49589
173	1	0.41424	-0.14292	-0.2726
174	1	0.35272	-0.65779	-0.27343
175	1	0.398966	-0.61762	-0.35073
176	1	0.316184	-0.63543	-0.1915
177	1	0.224673	-0.58307	-0.38467
178	1	0.38757	-0.41182	-0.03437
179	1	0.435936	-0.40446	-0.04026
180	1	0.605028	-0.60968	-0.32639
181	1	0.372357	-0.2935	-0.06504
182	1	0.371043	-0.62373	-0.23641
183	1	0.393424	-0.44733	-0.06092
184	1	0.250323	-0.60584	-0.2113
185	1	0.393899	-0.42112	-0.05912
186	1	0.397611	-0.28997	-0.08817
187	1	0.356246	-0.59621	-0.27212
188	1	0.396373	-0.19834	-0.17455
189	1	0.338449	-0.32139	-0.41979
190	1	0.720932	-0.52634	-0.11804
191	1	0.264862	-0.41648	-0.07269
192	1	0.252092	-0.47984	-0.09426
193	1	0.336272	-0.36517	-0.41758
194	1	0.417483	-0.46818	-0.09197
195	1	0.388154	-0.28625	-0.1093
196	1	0.332839	-0.56999	-0.2785

Serial	cluster	EndF	FAC1_2	FAC2_2
197	1	0.378418	-0.21593	-0.18763
198	1	0.314939	-0.2821	-0.37712
199	1	0.363475	-0.5192	-0.13957
200	1	0.246092	-0.55108	-0.27024
201	1	0.433681	-0.23514	-0.18854
202	1	0.355468	-0.54097	-0.29118
203	1	0.366623	-0.37959	-0.10404
204	1	0.367934	-0.37921	-0.1046
205	1	0.431319	-0.22868	-0.24474
206	1	0.314696	-0.48762	-0.33668
207	1	0.328961	-0.52773	-0.19145
208	1	0.343149	-0.25804	-0.3208
209	1	0.381798	-0.45031	-0.12626
210	1	0.351971	-0.53078	-0.22556
211	1	0.425861	-0.38166	-0.11656
212	1	0.340531	-0.52952	-0.2525
213	1	0.335398	-0.36181	-0.1307
214	1	0.345609	-0.33766	-0.14142
215	1	0.293475	-0.2804	-0.29975
216	1	0.33192	-0.40418	-0.33956
217	1	0.322251	-0.4936	-0.28015
218	1	0.339102	-0.31096	-0.32275
219	1	0.338356	-0.29989	-0.31169
220	1	0.380272	-0.44919	-0.15473
221	1	0.272069	-0.34745	-0.14774
222	1	0.386209	-0.49526	-0.21229
223	1	0.412838	-0.28361	-0.25738
224	1	0.381413	-0.33661	-0.3006
225	1	0.358381	-0.4454	-0.26402
226	1	0.351565	-0.40843	-0.18037
227	1	0.310814	-0.38062	-0.18034
228	1	0.246237	-0.33969	-0.20852
229	1	0.315323	-0.40729	-0.18868
230	1	0.18076	-0.33353	-0.24284
231	1	0.328251	-0.33731	-0.25057
232	1	0.32618	-0.35494	-0.21126
233	1	0.327805	-0.41742	-0.23163
234	1	0.310185	-0.39078	-0.25582
235	1	0.356412	-0.37582	-0.25254
236	1	0.246898	-0.37512	-0.21487
237	1	0.39059	-0.40948	-0.227
238	1	0.355428	-0.39867	-0.23078
270	4	0.867995	2.256958	1.20334
269	4	0.94518	2.205851	0.855858
268	3	0.354084	1.638143	-0.53724
267	3	0.493375	1.609981	-0.34985
265	3	0.275747	2.222042	-0.54677
266	3	0.275747	2.222042	-0.54677
264	3	0.445188	2.009316	-0.80392

Serial	cluster	EndF	FAC1_2	FAC2_2
263	3	0.404531	2.163482	-0.20863
262	3	0.619159	1.969916	-0.12183
261	3	0.535724	1.71442	-0.12694
260	3	0.325688	2.019499	-0.11523
259	3	0.306428	1.398065	-0.54339
258	3	0.229508	2.351484	-0.32284
257	3	0.386469	1.450233	-0.23983
256	3	0.423845	1.68972	-0.08794
255	3	0.306072	2.480629	-0.64101
254	3	0.360511	1.528919	-0.11971
253	3	0.49245	1.568462	-0.0916
252	3	0.347352	2.304398	-0.19396
251	3	0.580563	1.35165	-0.25425
250	3	0.379961	1.24717	-0.32332
240	2	2.041102	-1.12646	2.052305
249	3	0.494378	2.206463	0.032638
248	3	0.40728	3.169921	-0.84255
239	2	1.416049	0.182696	1.714824
247	3	0.479949	2.07659	0.140584
246	3	0.611061	2.016866	0.210957
245	3	0.496835	2.706042	0.073311
244	3	0.433899	3.135122	-0.00242
243	3	0.601072	1.993962	0.358586
242	3	0.600661	1.777643	0.457046
241	3	0.760228	3.060039	0.556125
